# Partially oxidized DJ-1 inhibits α-synuclein nucleation and remodels mature α-synuclein fibrils in vitro

**DOI:** 10.1038/s42003-019-0644-7

**Published:** 2019-10-30

**Authors:** Roshan Kumar, Sanjay Kumar, Pranita Hanpude, Abhishek Kumar Singh, Tanu Johari, Sushanta Majumder, Tushar Kanti Maiti

**Affiliations:** 1 Functional Proteomics Laboratory, Regional Centre for Biotechnology, NCR Biotech Science Cluster, 3rd Milestone Gurgaon-Faridabad Expressway, Faridabad, 121001 India; 2 Manipal Academy of Higher Education, Manipal, Karnataka 576104 India

**Keywords:** Biophysical chemistry, Enzymes, Prions, Proteins, Structural biology

## Abstract

DJ-1 is a deglycase enzyme which exhibits a redox-sensitive chaperone-like activity. The partially oxidized state of DJ-1 is active in inhibiting the aggregation of α-synuclein, a key protein associated with Parkinson’s disease. The underlying molecular mechanism behind α-synuclein aggregation inhibition remains unknown. Here we report that the partially oxidized DJ-1 possesses an adhesive surface which sequesters α-synuclein monomers and blocks the early stages of α-synuclein aggregation and also restricts the elongation of α-synuclein fibrils. DJ-1 remodels mature α-synuclein fibrils into heterogeneous toxic oligomeric species. The remodeled fibers show loose surface topology due to a decrease in elastic modulus and disrupt membrane architecture, internalize easily and induce aberrant nitric oxide release. Our results provide a mechanism by which partially oxidized DJ-1 counteracts α-synuclein aggregation at initial stages of aggregation and provide evidence of a deleterious effect of remodeled α-synuclein species generated by partially oxidized DJ-1.

## Abstract (web summary)

Kumar et al investigate how the partially oxidized state of protein DJ-1 (DJ-1Pox) inhibits aggregation of α-synuclein, a hallmark of Parkinson’s disease. They find that DJ-1Pox show enhanced adhesive properties, sequesters α-synuclein monomers to prevent nucleation and remodels α-synuclein fibrils in vitro.

## Introduction

Chaperones are crucial components of protein quality control system, which contributes to synthesis, folding, trafficking, and turnover of proteins^1^. In addition to these established functions, molecular chaperones also play an important role in solublization of amyloidogenic proteins in initial stages of the aggregation pathway by preventing self-assembly of disease associated proteins into toxic oligomers^2^. Several possible mechanisms for such action have been proposed but the identification of specific molecular events associated with given protein-chaperone system is particularly a challenging task. It is now clear that molecular chaperones interact not only with monomeric, misfolded, or unfolded forms of proteins but also with a variety of aggregated intermediates and restrict aggregation process at different stages including primary nucleation, secondary nucleation, fiber elongation and fragmentation of mature fibrils. The heat shock protein DNAJB6 has shown to suppress toxic effects of amyloid proteins by inhibiting primary nucleation^3^. On the other hand, BRICHOS inhibits secondary nucleation of amyloid-β and small heat shock protein αβ-crystallin inhibits both secondary nucleation and elongation of amyloid-β^4,5^. There are many chaperones including small heat shock proteins, Hsp70, and Hsp104 that inhibit the monomeric or oligomeric state of α-synuclein and prevent fibrillation in vivo and in vitro^6–8^. A few chaperones also possess fibril dismantling ability that immediately contributes to pathophysiology by affecting the level of amyloids and oligomeric species linked to cell toxicity^9,10^. Hsp70 chaperone machinery along with DNAJB1, and Hsp110 family nucleotide exchange factor disassembles α-synuclein fibrils within a few minutes in an ATP dependent manner^11,12^. Rifampicin efficiently disaggregates preformed α-synuclein fibrils in a concentration-dependent manner^13^. (−) Epigallo catechin gallate (EGCG), a green tea polyphenol, also remodels mature α-synuclein fibrils and reduces cellular toxicity^14,15^. EGCG remodels mature fibers to large non-toxic amorphous aggregates without release of monomer or diffusible oligomers. The hydrophobic interaction between EGCG and beta rich mature fibers promotes the conformational changes followed by fiber remodeling. Recently it has also been shown that the oxidized EGCG forms covalent Schiff base adduct formation through lysine residue in mature fibers. The covalent modification of amyloids can cross-link fibers and prevent fiber fragmentation or dissociation to produce toxic oligomeric species. However, the amyloid disintegration mechanism of non-ATPase chaperones is still unclear and needs detailed investigation.

DJ-1 is a small ubiquitously expressed protein with a myriad of functions including redox sensing in oxidative stress, oxidative stress quenching, transcription regulation, chaperone, and glyoxalase function and it is involved in the glycation repair mechanism^16–19^. DJ-1 protein is a homodimer and each monomer consists of 189 residues, which structurally fold into seven beta-strands and eight helices homologous to members of ThiJ/PfpI family^20^. DJ-1 contains three cysteine residues including Cys46, Cys53, and Cys106 of which Cys106 is highly conserved^21^. Cys106 is buried deep in the putative binding site with a strained dihedral conformation. Lower pK_a_ of Cys106 (pKa ~ 5.4) makes this residue very reactive and thus sensitive to oxidation^22,23^. The side chain of Cys106 can be oxidized from thiolate (−S^−^) to sulfinate (−SO_2_^−^), or sulfonate (−SO_3_^−^)^24^. The mono oxidized, sulfenate (SO^−^) state of DJ-1 is assumed to be transient, while the un-oxidized and oxidized states are stable and have many distinct physiological functions. The oxidation state of Cys106 determines the specific functions of DJ-1. Particularly, the oxidation of Cys106 to the sulfinate state has neuroprotective functions^25^. The oxidative damage of DJ-1 due to over-oxidation is linked to PD and observed in post-mortem brain samples of PD and Alzheimer’s disease (AD) patients^26^. The impaired functions of over-oxidized DJ-1 play an important role in the onset and progression of PD. The environmental toxin like paraquat increases oxidized forms of DJ-1 as analysed using two-dimensional gel electrophoresis^27^. DJ-1 is expressed ubiquitously in many tissues including GI tract, pancreas, and brain^28,29^. However, it is widely distributed and highly expressed in brain^29^. Its localization in cytoplasm, mitochondria, and nucleus is recognized, but the relevance of this subcellular distribution to its cytoprotective activity is not fully understood. Mitochondrial localization of DJ-1 provides neuroprotective function^30^. Recent electron microscopy studies have demonstrated that DJ-1 is present both in mitochondrial matrix and inner membrane space^31,32^. DJ-1 knockdown studies in animal models show a decrease in mitochondrial complex activities, mitochondrial membrane potential and increased reactive oxygen species^33–35^ in the absence of DJ-1. Human DJ-1 in the presence of inorganic phosphate forms filamentous aggregates and it also forms fibrils under denaturing conditions^36^. DJ-1 chaperone activity is accessed towards the suppression of heat-induced aggregation of citrate synthase (CS) and glutathione S-transferase (GST)^37^. It also activates SOD1 through copper transfer and plays an important role in redox quenching^38^. However, recent NMR experiments in cells have demonstrated that DJ-1 does not carry any metal ions in any oxidation state and copper transfer from DJ-1 to SOD1 also appears controversial^38^. In this regard more studies are necessary to address this issue. The chaperone activity of DJ-1 is abolished under reducing conditions suggesting that DJ-1 is a redox-regulated chaperone that is active only in an oxidizing environment^39^. Recently it has also been shown that partially oxidized form of DJ-1 inhibits α-synuclein aggregation^24,37^. How only partially oxidized form of DJ-1 is active towards α-synuclein fibrillation inhibition compared to un-oxidized and hyper-oxidized forms needs detailed investigation.

In the present study, we report that differential adhesive surfaces in diverse forms of DJ-1 are indeed an important element of its amyloid aggregation inhibition mechanism. The enhanced adhesive property of partially oxidized form of DJ-1 prevents α-synuclein nucleation and elongation. The partially oxidized form of DJ-1 strongly binds mature α-synuclein fibrils relative to other forms of DJ-1. The remodeling property of partially oxidized DJ-1 towards mature α-synuclein fibrils is due to preferential binding, fibril surface wrapping and outward pulling force. The remodeled fibrils generate toxic oligomeric species, which induce cellular membrane perturbations, actin destabilization, altered nitric oxide release, and apoptotic cell death.

## Results

### Partially oxidized DJ-1 posesses strong adhesive properties

**Fig. 1:**
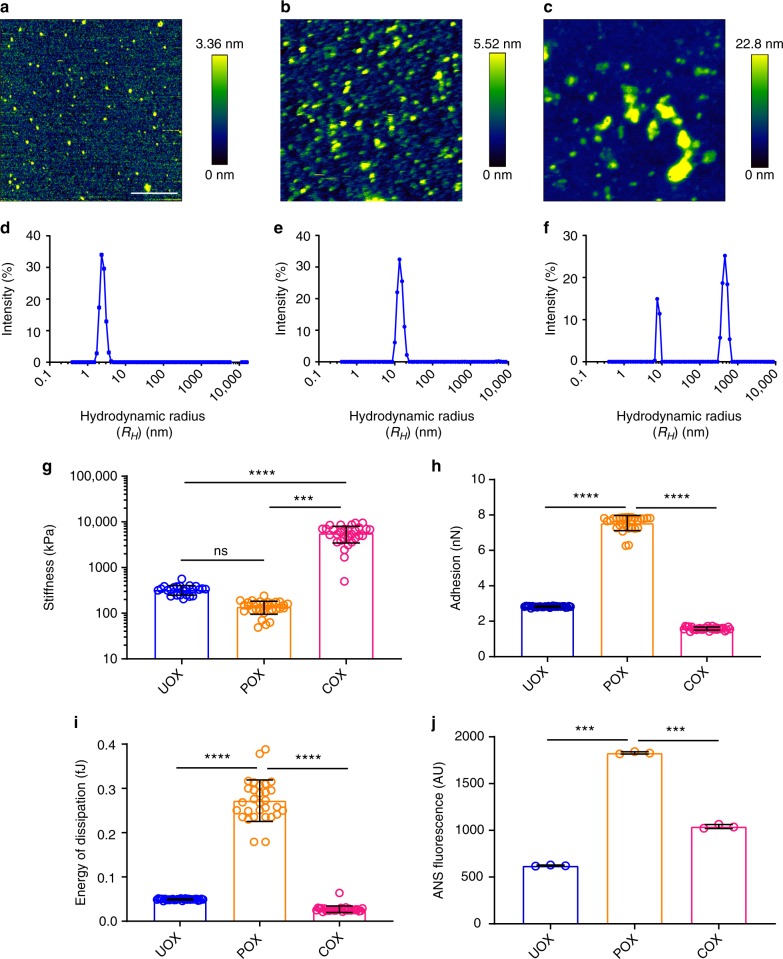
Mechanical properties of different forms of chaperone DJ-1. QI AFM images (2 × 2 μm) showing morphological characteristics of **a** DJ-1_Uox_, **b** DJ-1_Pox_ and **c** DJ-1_Cox_. **d**–**f** 1 mg/ml of different forms of DJ-1 were used for DLS measurement. **g** Young’s modulus, **h** adhesion, **i** energy of dissipation and **j** ANS dye-binding assay. The parameters are shown in the image **d**, **e**, and **f** which were measured from three different experiments and plotted ~28 points for each form of DJ-1 from three biologically independent experiments. **P, ***P, and ****P < 0.1, analyzed using one-way ANOVA with Bonferroni’s post-hoc test. Scale bar represents 500 nm

Human DJ-1 comprises of three cysteines (Cys106, Cys53, and Cys46) and six methionine. Under hyper-oxidized conditions in vitro, all of these residues undergo oxidation and produce aggregated species of DJ-1. However, under controlled oxidation conditions, DJ-1 produces Cys106-SO_2_^−^ (we named it partially oxidized or DJ-1_Pox_). Under strong oxidative conditions, Cys106 undergoes oxidative modification and generates Cys106-SO_3_^−^ species (thereafter we named it as completely oxidized or DJ-1_Cox_). It has been demonstrated that DJ-1_Pox_ shows chaperone function and inhibits the aggregation of α-synuclein. α-Synuclein aggregation inhibition by DJ-1 might work through inhibition of nucleation or fiber elongation processes or disassembly of fibers. The precise mode of aggregation inhibition by DJ-1 is still unclear. DJ-1_Pox_ and DJ-1_Cox_ species have been made in in vitro condition based on the published protocol^24^. DJ-1_Pox_ and DJ-1_Cox_ species have been confirmed by MALDI-MS/MS analysis (Supplementrary Fig. 1 and Supplementary Table 1). In the present study, we used C-terminal His-tagged DJ-1 without removal of the histidine tag from the protein. It has been demonstrated earlier that the histidine tag does not affect the chaperone activity of DJ-1^37^. We also purified untagged DJ-1 and made different species of untagged DJ-1 (untagged DJ-1_Uox_ and untagged DJ-1_Pox_). Un-oxidized (untagged) and Partially oxidized (untagged) DJ-1 was confirmed by MALDI-TOF analysis (Supplementary Fig. 2a, b). Both His-tag DJ-1_Pox_ and untagged DJ-1_Pox_ showed similar aggregation inhibition of α-synuclein. (Supplementary Fig. 2e). Similarly, untagged DJ-1 and His-tag DJ-1 also showed similar deglycase activity having K_m_ 0.014 and k_cat_ 0.01 (Supplementary Fig. 2c, d and Supplementary Table 2).

Far-UV circular dichroism (CD) spectral analysis showed a minor structural change in DJ-1_Pox_ compared to DJ-1_Uox_ (Supplementary Fig. 3). The different forms of DJ-1 were viewed in atomic force microscopy to understand its morphological features. DJ-1_Uox_, DJ-1_Pox,_ and DJ-1_Cox_ showed globular oligomers with a height profile of ≤3.36 nm ≤5.52 nm and ≤22.8 nm, respectively (Fig. 1a–c). The dynamic light scattering (DLS) experiments showed a presence of species with a hydrodynamic radius of ~2.33 nm ~13.5 nm and heterogeneous populations of 7.53 nm and 459 nm for DJ-1_Uox_, DJ-1_Pox_ and DJ-1_Cox_, respectively (Fig. 1d–f). Circular dichroism results along with DLS data have confirmed that DJ-1_Pox_ formed a specific oligomeric species where the structure is unperturbed. Force spectroscopy measurement was carried out to understand the nanomechanical properties of different forms of DJ-1. DJ-1_Uox_, DJ-1_Pox,_ and DJ-1_Cox_ showed Young’s modulus of 466 ± 96 kPa, 153 ± 23 kPa, and 6690 ± 1378 kPa, respectively (Fig. 1g). Young’s modulus of protein is inversely related to its adhesive property. We extracted the surface adhesive parameters from force spectra and they were found to be 2 ± 0.1 nN, 8 ± 0.5 nN and 1 ± 0.1 nN for DJ-1_Uox_, DJ-1_Pox_ and DJ-1_Cox_, respectively (Fig. 1h). To corroborate the adhesive surface of DJ-1_Pox_ with the surface hydrophobicity, l-Anilinonaphthalene-8-sulphonate (ANS) binding experiment was performed. The fluorescence intensity of ANS increased significantly after binding to the DJ-1_Pox_ compared to DJ-1_Uox_ and DJ-1_Cox_ (Fig. 1j). All these results confirmed that DJ-1_Pox_ produces oligomers with the strong adhesive property.

### DJ-1_Pox_ inhibits nucleation of α-synuclein

**Fig. 2:**
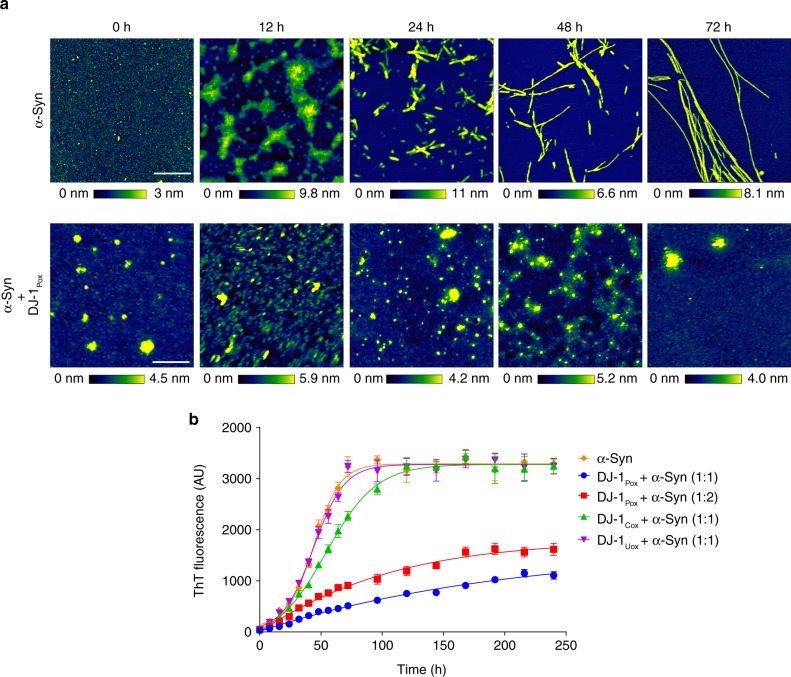
DJ-1_Pox_ inhibits nucleation of α-synuclein. **a** Time-dependent visualization of α-synuclein aggregation inhibition in presence of DJ-1_Pox_. The top panel represents normal aggregation pattern followed by α-synuclein (200 μM) and bottom panel represents amorphous like species when α-synuclein (200 μM) is co-incubated with DJ-1(200 μM). **b** aggregation kinetics of α-synuclein with and without different forms of DJ-1 measured using Thioflavin T assay. Scale bar represents 500 nm

Inhibition of α-synuclein aggregation by DJ-1_Pox_ has been studied in in vitro and in cell culture^24,37^. However, the mode of inhibition has not been elucidated. To confirm the inhibitory effect of DJ-1_Pox_ on α-synuclein aggregation, fibrillation kinetics of α-synuclein was monitored in the presence and absence of different forms of DJ-1 using Thioflavin T (ThT) dye. Incubation of DJ-1_Pox_ with low molecular weight species of α-synuclein showed inhibition of fibrillization of α-synuclein in a time-dependent manner (Fig. 2a). The aggregation rate constant and t_lag_ of α-synuclein aggregation in the presence of different forms of DJ-1 were presented in the (Supplementary Table 3). The t_lag_ of α-synuclein, α-synuclein with DJ-1_Pox_ (1:1), α-synuclein with DJ-1_Pox_ (1:2), α-synuclein with DJ-1_Uox_ (1:1) and α-synuclein with DJ-1_Cox_ (1:1) were 27.6 ± 0.9 h, 35.1 ± 1.6 h, 25.8 ± 1.9 h, 31.5 ± 0.6 h, and 27.2 ± 0.8 h, respectively. The aggregation rate constant (K_app_) of α-synuclein, α-synuclein with DJ-1_Pox_ (1:1), α-synuclein with DJ-1_Pox_ (1:2), α-synuclein with DJ-1_Uox_ (1:1) and α-synuclein with DJ-1_Cox_ (1:1) were 0.055 ± 0.006 h^−1,^ 0.016 ± 0.0003 h^−1^, 0.023 ± 0.001 h^−1^, 0.034 ± 0.002 h^−1^, and 0.051 ± 0.0012 h^−1^, respectively (Supplementary Table 3). To validate the inhibition of α-synuclein aggregation by DJ-1_Pox_, time-dependent AFM studies had been carried out at 0, 12, 24, 48, and 72 h. α-Synuclein showed the evolution of different stages of aggregates (oligomeric aggregates, proto-fibrils, and mature fibrils) in absence of DJ-1_Pox_ (Fig. 2b). However, co-incubation of α-synuclein with DJ-1_Pox_ produced only amorphous aggregates at all time points. The reproducibility of DJ-1_Pox_ in α-synuclein nucleation inhibition was also tested (Supplementary Fig. 4). Thus, our ThT and AFM results demonstrated that α-synuclein aggregation inhibition was mediated through the inhibition of nucleation phase.

### DJ-1_Pox_ inhibits elongation of α-synuclein fibrillation

**Fig. 3:**
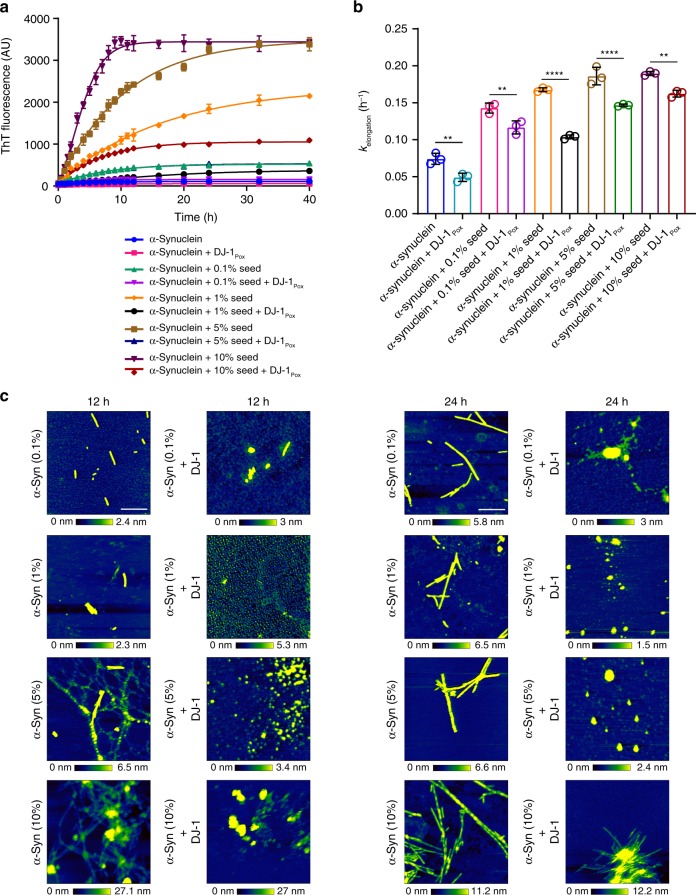
Seeded aggregation of α-synuclein in presence of DJ-1_Pox_. **a** Increase of relative aggregate mass upon addition of free monomers (50 µM) of α-synuclein to existing seeds (0.1, 1, 5, and 10%) was measured over time by Thioflavin T fluorescence assay. **b** Elongation rate of α-synuclein seeds in presence of α-synuclein monomers as a function of DJ-1_Pox_. From three biologically independent experiments. **P, ***P, and ****P < 0.1, analyzed using one-way ANOVA with Bonferroni’s post-hoc test. **c** AFM images showing seed elongation and inhibition in presence of DJ-1_Pox_ at different time points. Scale bar represents 500 nm

Addition of preformed truncated short fibril of α-synuclein to the low molecular weight (LMW) species of α-synuclein causes acceleration of fibrillation and it has been demonstrated in many literatures that the fibrillation proceeds through elongation mechanism. Inhibition of aggregation of α-synuclein by chaperones or small molecules might also occur through elongation inhibition mechanism. To find out whether DJ-1 also inhibits the elongation of α-synuclein fibrils, a seeding reaction was performed using a varying amount of α-synuclein seeds ranging from 0.1 to 10%. The initial aggregation kinetics of seeded reaction involves elongation of existing seeds due to monomer addition. The increase of fibril mass upon fibril elongation was monitored by ThT fluorescence. Indeed, the aggregation kinetics of α-synuclein was markedly slower in the presence of only DJ-1_Pox_ (Fig. 3a and Supplementary Fig. 5). The relative elongation rate constants (k) of α-synuclein were then derived from the linear fits of the initial aggregation kinetics. The elongation rates varied with the concentration of α-synuclein seeds (0.1–10%) and incubation of DJ-1_Pox_ significantly inhibited elongation rates in all seeding conditions (Fig. 3b). On the other hand, neither un-oxidized DJ-1 nor completely oxidized DJ-1 showed inhibition of seeded aggregation even at higher concentration. Time-dependent AFM experiments were performed at 0, 24, 48, and 72 h to visualize the elongation inhibition by DJ-1_Pox_. The amorphous aggregates were seen at different time points when DJ-1_Pox_ was co-incubated with α-synuclein along with different concentrations of seeds (Fig. 3c). However, in presence of 10% α- synuclein seed with DJ-1_Pox,_ α- synuclein showed some fibrillary structure at 72 h. Seeded aggregation of α-synuclein in the presence of un-oxidized DJ-1 and completely oxidized DJ-1 showed fibril elongation in AFM studies (data not shown). Our data demonstrated that DJ-1_Pox_ not only inhibits nucleation of aggregation processes but also inhibits elongation processes of amyloid fiber formation.

### DJ-1_Pox_ binds with mature fibers and remodels them in vitro

**Fig. 4:**
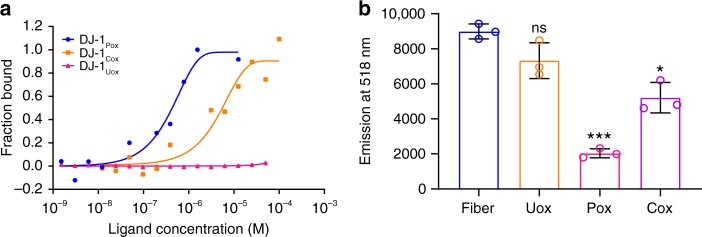
Remodeling of mature α-synuclein fibrils in the presence of DJ-1_Pox_. **a** Microscale thermophoresis (MST) was performed to measure the binding of DJ-1 to α-synuclein mature fibrils. **b** Fiber disintegration assay with different forms of DJ-1

**Fig. 5:**
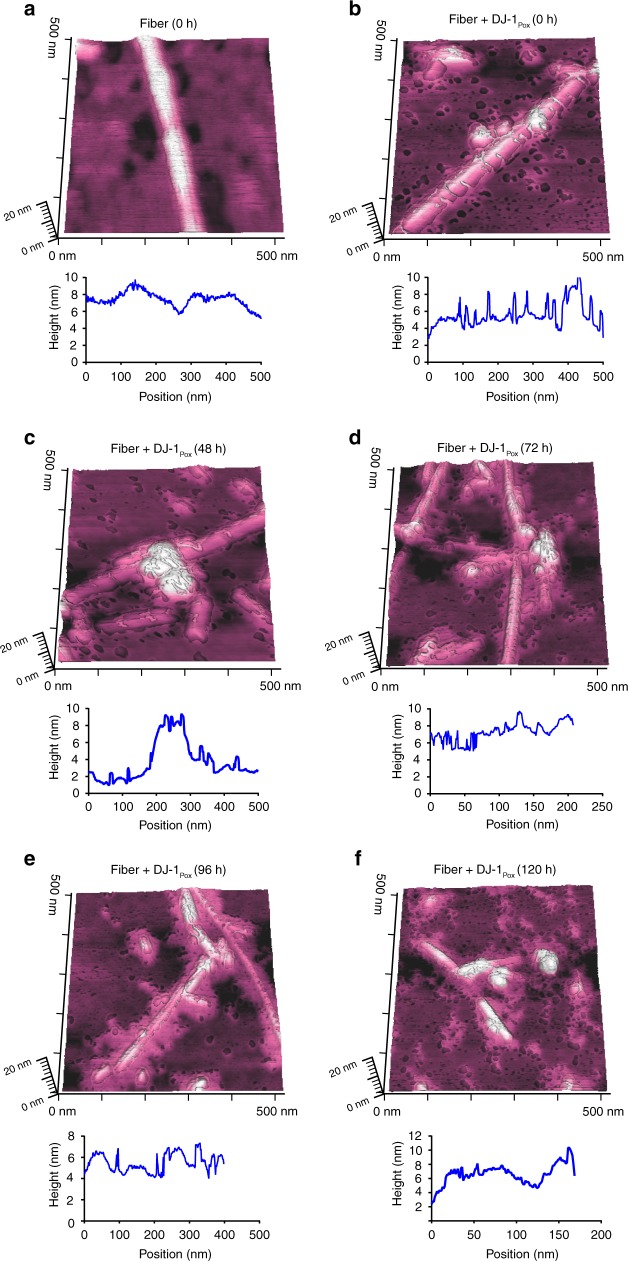
Remodeling of α-synuclein at single fibril resolution. AFM images showing α-synuclein mature fibrils remodeling by DJ-1_Pox_. **a** α-Synuclein mature fibril. **b** Fiber + DJ-1_Pox_ at 0 h. **c** Fiber + DJ-1_Pox_ at 48 h. **d** Fiber + DJ-1_Pox_ at 72 h. **e** Fiber + DJ-1_Pox_ at 96 h. **f** Fiber + DJ-1_Pox_ at 120 h. Line profile of single fiber is shown at the bottom of AFM image

**Fig. 6:**
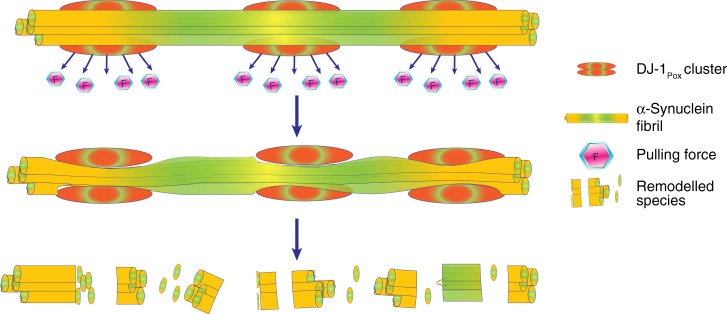
Remodeling of α-synuclein at single fibril resolution. Schematic representation showing α-synuclein mature fibril disintegration by DJ-1_Pox_. DJ-1_Pox_ wraps around the α-synuclein mature fibrils, which creates an outward pulling force that makes the fiber thinner and ultimately converts it to shorter oligomeric species

**Fig. 7:**
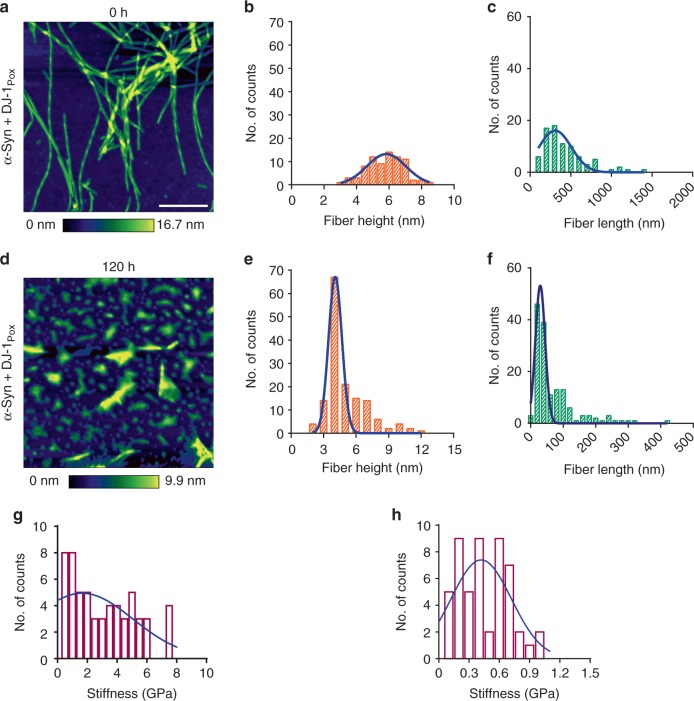
Property of remodeled fibrils. **a** α-synuclein mature fibrils. Gaussian distribution of α-synuclein fibrils **b** height and **c** length. α-synuclein mature fibrils with DJ-1_Pox_ **d** AFM, **e** height, **f** length, stiffness of **g** α-synuclein fibrils and **h** α-synuclein fibrils with DJ-1_Pox_. Scale bar represents 500 nm

Amyloid fiber disassembly or fiber remodeling is one of the important mechanisms by which chaperones regulate protein homeostasis. Typically, heat shock proteins of large dynamic oligomers like Hsp70, Hsp90, and Hsp104 function as ATP dependent chaperones that bind to mature fibers and disassemble the fibers to soluble species. The mechanism of fiber disassembly has also been elucidated in the literature. However, disaggregase function of small heat shock proteins has not been studied thoroughly. Recently, the amyloid depolymerisation of yeast prion protein Sup35 by yeast heat shock proteins Hsp26 and Hsp42 was documented^40^. The binding of α-synuclein mature fibers with different forms of DJ-1 was performed using Microscale Thermophoresis (MST). The binding constants of α-synuclein fibrils with DJ-1_Uox_, DJ-1_Pox_ and DJ-1_Cox_ were 1965 ± 6 µM, 0.16 ± 0.1 µM, and 4.8 ± 2.1 µM, respectively (Fig. 4a). The binding data demonstrated that DJ-1_Pox_ binds almost 25 times higher than fully DJ-1_Cox_. We also tested the remodeling capabilities of different forms of DJ-1 by co-incubating with FITC labelled mature α-synuclein fibrils. Different form of DJ-1 species showed significant α-synuclein leaching from mature fibers and this leaching was maximum in case of DJ-1_Pox_ treatment condition and almost nonsignificant effect was observed in case of DJ-1_Uox_ condition (Fig. 4b). The effect of the different oxidized forms of DJ-1 was also monitored by time-dependent AFM and force spectroscopy. We found that the incubation of mature α-synuclein fibrils with DJ-1_Pox_ remodeled fibers through cascade of steps. At 0 h mature α-synuclein fibrils were smooth in texture with a rod-like appearance in 3D visualization. At similar time point, α-synuclein fibrils with DJ-1_Pox_ showed wrapping of fibril and presence of globular molecule. These globular molecular structures might be due to the accumulation of DJ-1 on fibril surface. Accumulation of DJ-1_Pox_ over fibril surface was observed at 48 h and a nick was created at 72 h. The surface topology of fibril showed as a corroded fiber at 96 h and around 120 h, fragmented protofibrillar as well as small oligomeric structures were visible in the AFM images (Fig. 5 and Supplementary Fig. 6). The schematic of α-synuclein fibril disintegration was shown in Fig. 6. The transverse line profile on a single fiber of α-synuclein in the absence of DJ-1_Pox_ showed a periodicity of α-synuclein arrangement and this was ~150–300 nm. However, DJ-1_Pox_ alters the periodicity of α-synuclein arrangement on longer time incubation. The average height and length of native and remodeled fibrils of α-synuclein were measured. The height and length of the native fibrils were 6 nm, 450 nm, respectively, as reported in the literature (Fig. 7b, c). On the other hand, remodeled species showed both changes in height and length and they were ~3.5 nm and 60 nm, respectively (Fig. 7e, f). Packing of the α-synuclein monomeric unit within the fiber provides the fiber strength. Remodeling of fiber removes the α-synuclein unit from the ordered fiber surface and reduces fiber strength. The Young’s modulus that represents strength of mature fibrils was 3 GPa as reported earlier. However, Young’s modulus of remodeled species was significantly lower than mature fibrils and it was ~0.6 GPa (Fig. 7g, h). DJ-1_Uox_ and DJ-1_Cox_ were not efficient in disintegrating α-synuclein fibrils (Supplementary Fig. 7).

### Remodeled fibers are more toxic than mature fibrils

**Fig. 8:**
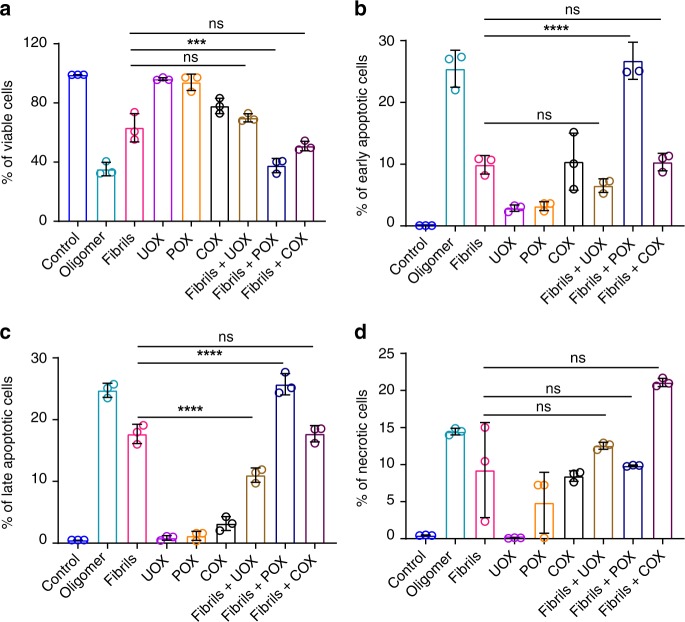
Different degrees of neurotoxicity induced by α-synuclein variants in presence and absence of DJ-1_Pox_ SH-SY5Y cells were treated with 10 µM of different α-synuclein oligomers and monomers for 48 h. Cells were then detached using 1X PBS and 5 mM EDTA and apoptosis assay was performed using Annexin V-FITC Apoptosis Detection Kit (Sigma, USA). Different samples (1 × 10^6^ cells/ml) were treated with Annexin V- FITC (5 µl) and PI (10 µl) and incubated at room temperature for exactly 10 min in dark. Labelled cells were sorted by fluorescence using a BD FACS verse Flow Cytometer (BD Biosciences) with a minimum of 10,000 events recorded per sample using BD Cell Quest Pro software. Three biologically independent experiments were performed. Statistical significance was analyzed using one-way ANOVA with Bonferroni’s post hoc test (**P, ***P, and ****P < 0.1). Panel represents percentage of **a** viable cells, **b** early apoptotic cells, **c** late apoptotic cells and **d** necrotic cells

We performed Annexin V/PI staining followed by FACS analysis to test the toxic behavior of remodeled species and found that the remodeled fibrils were more toxic than mature fibrils towards retinoic acid differentiated SH-SY5Y cells. The percentages of viable cells in response to different oligomeric preparations are shown in (Fig. 8a). The viable cells in response to LMW, oligomer, fibrils, DJ-1_Uox,_ DJ-1_Pox,_ DJ-1_Cox_, fibrils with DJ-1_Uox,_ fibrils with DJ-1_Pox_, fibrils with DJ-1_Cox_ were 99, 35, 63, 96, 94, 78, 70, 38, and 51%, respectively. The percentages of early apoptotic cells in response to monomers, oligomer, fibrils, DJ-1_Uox,_ DJ-1_Pox,_ DJ-1_Cox,_ fibrils with DJ-1_Uox,_ fibrils with DJ-1_Pox_, fibrils with DJ-1_Cox_ were <1, 25, 10, 3, 3, 10, 7, 26 and 10%, respectively. The percentages of late apoptotic cells in response to LMW, oligomer, fibrils, DJ-1_Uox,_ DJ-1_Pox,_ DJ-1_Cox,_ fibrils with DJ-1_Uox,_ fibrils with DJ-1_Pox_, fibrils with DJ-1_Cox_ were <1, 25, 18, <1, 1, 3, 11, 26, and 18%, respectively. The percentages of necrotic cells in response to LMW, oligomer, fibrils, DJ-1_Uox,_ DJ-1_Pox,_ DJ-1_Cox,_ fibrils with DJ-1_Uox,_ fibrils with DJ-1_Pox_, fibrils with DJ-1_Cox_ were <1, 14, 9, <1, 5, 8, 13, 10, and 21%, respectively (Fig. 8b–d).

### Mechanism of neurotoxicity of remodeled fibrils

**Fig. 9:**
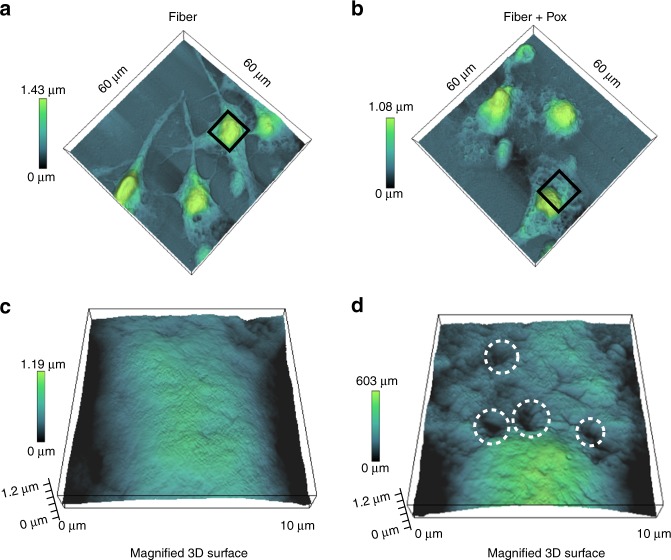
Differential membrane architecture alteration in the presence of α-synuclein fibrils derived oligomeric conformations. 3D atomic force microscopic images (60 × 60 µm) of SH-SY5Y cells. A magnified 3D surface (10 × 10 µm). **a**, **c** Fibrils treated. **b**, **d** Fibrils derived α-synuclein oligomer treated. The membrane damage is marked with a white circle. Each image is presented with a height scale. Distinct pore-like features and neurites degeneration were clearly seen on neuroblastoma SH-SY5Y cells treated with fibrils derived α-synuclein oligomers. The neurite degeneration is absent in fibrils treated cells. Certain membrane perturbations were also seen with fibril treated cells

To understand the mechanism of neurotoxicity by remodeled fibrils of α-synuclein, SH-SY5Y cells were incubated with 10 μM of remodeled as well as intact fibrils for 48 h. High-resolution AFM images were taken from control and treated SH-SY5Y cells in low force contact mode. Representative images of SH-SY5Y cells upon treatment of BSA (control), different α-synuclein species and remodeled fiber species at 48 h are shown in Fig. 9. The SH-SY5Y cells treated with BSA and DJ-1_Pox_ showed smooth cell surface of conventional epithelial-like shape with distinct boundaries, centrally located nuclei and neurites like projections (Supplementary Fig. 8). α-Synuclein fibrils (10 µM) treatment to SH-SY5Y cells revealed uneven cell surface with a few pores (Fig. 9a, c). However, cells treated with remodeled species displayed degenerated neurites and constriction of the cytoplasmic region. A mixed population of small and large-sized pores were seen at nanoscale resolution along with rougher surface after treatment with remodeled fiber (Fig. 9b, d). The cytoskeletal protein actin forms filaments that provide mechanical support to cytoskeletal network. To understand the mechanism behind this membrane surface alteration we probed the change in actin filaments of SH-SY5Y cells upon treatment with remodeled fibrils. The distinct actin spikes were also seen in BSA treated cells (Supplementary Fig. 9). The retraction of actin to the perinuclear region was seen in cells treated with remodeled fibril (Supplementary Fig. 9). On the other hand, oligomers treated with DJ-1_Pox_ maintained cytoskeletal integrity (Supplementary Fig. 9).

It is also well established that short or truncated fibrils of α-synuclein show a prion-like behavior and translocate from one cell to another through cellular internalization. However, longer mature fibrils have poor internalization capabilities. To test whether remodeled fibrils also possess an internalization tendency, we treated SH-SY5Y cells with FITC labelled remodeled fibrils, FITC- mature fibrils, and FITC- DJ-1_Pox_ for 30 min. We found that the remodeled fibrils were internalized more efficiently than mature fibrils and DJ-1_Pox_ (Supplementary Fig. 10a). This observation confirmed that DJ-1_Pox_ remodeled fibrils are shorter species. Our earlier report has demonstrated that extracellular α-synuclein also induces neurotoxicity due to increase in cellular nitric oxide level and subsequent protein S-nitrosylation^41^. Here we found that remodeled fibrils induced an almost two-fold higher nitric oxide level than mature fibrils in SH-SY5Y cells (Supplementary Fig. 10b).

## Discussion

The aggregation of an amyloidogenic protein proceeds through generation of either an on- or off-folding species pathway, which ultimately produces protein aggregates. It has been demonstrated that the on-pathway species are toxic whereas off-pathway species are irreversible and non-toxic^42^. Chaperones have an inherent property to interact with on pathway species and neutralise its toxicity^43^. Here we have demonstrated that DJ-1 in the partially oxidized state generates functional oligomeric species, which has strong adhesive properties, reduced stiffness, and an exposed hydrophobic surface. The structural evidence supports the presence of a Cys106-SO_2_^−^ moiety in the crystal. One important observation in previous structural studies is that DJ-1-Cys106 residue undergoes oxidation during the crystallization process^44^. This modification is retained in the crystal and the overall structure of DJ-1 Cys106-SO_2_^−^ is very similar to the un-oxidized condition. In contrast, in vitro controlled oxidation by hydrogen peroxide in solution produces an oligomeric assembly. This can be explained by the fact that crystallization is a slow process where this oxidation happens slower in this residue. However, hydrogen peroxide-mediated controlled oxidation is kinetically a rapid processes where the protein is driven towards self-assembly. These adhesive oligomers act as a trap, which sequesters α-synuclein monomers and blocks the early stages of α-synuclein aggregation by suppressing the formation of α-synuclein nuclei^45,46^.

Seeded aggregation is one of the characteristic mechanisms of fibrillation for all amyloid proteins and amplification of amyloid aggregates occurs rapidly through this mechanism. Seeded aggregation initiates through template-dependent and independent processes. The elongation of fiber happens by attachment of monomers through the tip of truncated fibrils and it is template-dependent. On the other hand, the monomeric molecule also catalyzes the nucleation on the surface of truncated fibrils and perpetuates the protein conformation like core fibrils and it is template independent.^47^. Our present study demonstrates that DJ-1_Pox_ inhibits α-synuclein seeded aggregation, alters the α-synuclein aggregation network and restricts the production of toxic oligomeric on-pathway species. It is known that in the presence of excessive oxidants, Cysteine 53 becomes oxidized at the dimer interface and this destabilises the homodimer of DJ-1 leading to the formation of amorphous aggregates^25^. Here we show that the enhanced surface adhesive property of DJ-1_Pox_ makes it more efficient in controlling α-synuclein aggregation compared to its un-oxidized and completely oxidized or hyper-oxidized counterparts. The binding studies of different forms of DJ-1 with mature α-synuclein fibers describe the preferential recruitment of DJ-1_Pox_ on the fiber surface in vitro. Our data provide strong support for the hypothesis that surface adhesive property of DJ-1_Pox_ dictates its preferential binding with α-synuclein mature fibrils.

Amyloid fiber disintegration and remodeling are mechanisms by which many chaperones like Hsp70 and Hsp104 control the cytotoxicity of fibrils^8,48^. The ATP dependent chaperones bind on to the fiber surface and remove the monomeric unit by pulling force. The energy generated due to the hydrolysis of ATP facilitates the fiber disassembly process. Thus, ATP dependent chaperones disassemble the fibers rapidly than non-ATP dependent chaperones. Therefore, we also tested if DJ-1_Pox_ can disassemble or remodel the mature α-synuclein fibers. Interestingly, DJ-1_Pox_ remodels the mature α-synuclein fibrils by strong interaction with the fibers. In the initial phase, mature synuclein recruits DJ-1_Pox_ to the surface and quickly wraps the fiber surface that has appeared in 3D AFM image. The wrapping of DJ-1 may generate strong pulling forces which slowly truncates mature fibrils. The leaching of α-synuclein fibrils makes fibril structure thinner with loss of characteristic fibrillar periodicity. An alternative explanation for the remodeling of the α-synuclein fiber is that the partial rupture of filament may happen due to collision/bending/Brownian motion of DJ-1_Pox_ and fibers during their interaction. DJ-1 caps and inserts in the α-synuclein fibril resulting in the fragmentation of the fibrils.

We have characterized the remodeled species in terms of dimensions and elasticity. The size and Young’s modulus of remodeled species are very similar to oligomeric species of α-synuclein. The remodeled species show similar neurite degeneration, cellular perforation, membrane damage and cytoskeletal alteration as observed in the oligomeric α-synuclein exposure to cells. The extracellular α-synuclein oligomeric species, as well as short truncated fibrils, internalize in the cells via endocytic and non-endocytic mechanism^49,50^. Similar to oligomeric species, the remodeled fibers also efficiently internalize in the cells. Our results provide strong evidence that DJ-1_Pox_ remodeled fibrils behave as oligomeric species. We have so far been unable to demonstrate a specific effect of partially oxidized DJ-1 in the remodeling of α-synuclein in cells. Finally, our data have demonstrated that the remodeled fibers also induce ROS/RNS species, which eventually promotes cell death.

In short, our results have demonstrated that DJ-1_Pox_ is active chaperone that inhibits primary nucleation and elongation in α-synuclein aggregation cascade. The adhesive property of DJ-1_Pox_ is an important parameter which regulates its anti-aggregation activity. DJ-1_Pox_ also remodels fibers, generates toxic oligomeric species and induces neuronal cell death. Thus, our results not only provide novel insights into the molecular mechanisms by which the DJ-1 counteracts with the α-synuclein aggregation at initial stages of aggregation but also provides a detrimental mechanism of its chaperone function.

## Methods

### Expression and purification DJ-1 and α-synuclein

pET3a-His-DJ1 (Plasmid 51488, Addgene) was a gift from the Michael J Fox Foundation (MJFF). BL 21 (DE3) *E. coli* cells containing pET3a-His-DJ1 plasmid were grown in LB media in presence of 100 µg/ml of ampicillin at 37 °C. Cells were induced with 0.1 mM IPTG at 0.7 OD and incubated overnight at 20 °C with 180 rpm shaking. The cells were harvested by centrifugation and the pellet was stored at −80 °C until needed. Pellet was resuspended in lysis buffer containing 50 mM Tris, pH 8.0, 100 mM NaCl, 1 mM PMSF with streptomycin sulphate (10 mg/ml) and lysozyme and the resuspended cells were kept at 4 °C for 20 min. The cells were homogenized by probe sonicator with 70% amplitude and 10 pulses/min for 10 min and centrifuged at 16,000 × *g* for 1 h at 4 °C. The supernatant was loaded on to Ni^2+^-NTA column which was pre-equilibrated with 50 mM Tris, pH 8.0, 100 mM NaCl, 5 mM Imidazole at 4 °C. The column was washed with 100 ml of equilibration buffer and the protein was eluted with 50 mM Tris, pH 8.0, 50 mM NaCl, 300 mM Imidazole. Eluted DJ-1 protein was dialyzed against dialysis buffer containing 10 mM Tris, pH 8.0, 50 mM NaCl, 1 mM DTT at 4 °C. The protein was concentrated and loaded on to Hiload 16/600 Superdex 75 gel filtration column. The purity of the protein was checked in SDS-PAGE. Protein concentration was measured using molar extinction coefficient of monomeric DJ-1 4200 M^−1^ cm^−1^.

pT7-7 α-Synuclein (Plasmid 36046, Addgene) was expressed in *E. coli* BL21 (DE3) strain using established protocol. Briefly, IPTG induced cells were harvested by centrifugation (6000 × *g*, 30 min). The pellet was resuspended in lysis buffer (50 mM Tris, pH 8.0, 10 mM EDTA, 150 mM NaCl, 1 mM PMSF). Sonication was carried out using probe sonicator at 50% amplitude and 45 pulses/min for 10 min at 4 °C. Cell lysate was heated at 95 °C for 20 min and it was cooled to room temperature. The cell lysate was centrifuged at 14,000 × *g* for 30 min. Streptomycin sulphate (10 mg/ml) and glacial acetic acid (228 µl/ml of supernatant) were added to the supernatant and it was kept at 4 °C for 30 min. The solution was then centrifuged again at 14,000 × *g* for 30 min to remove aggregated proteins. The clarified protein solution was precipitated with ammonium sulphate of final concentration of 36% for 1 h at 4 °C. The precipitated protein was suspended in 100 mM ammonium acetate and further precipitated by adding an equal volume of absolute ethanol. This precipitation step was repeated twice. Finally, protein pellet was suspended in 100 mM ammonium acetate and solution was lyophilized and stored at −20 °C for further use^51^.

### Differential oxidation of DJ-1 and characterisation using MALDI MS

The oxidation of DJ-1 was performed by incubating DJ-1 with different concentrations of hydrogen peroxide (H_2_O_2_) as described earlier with slight modifications^24^. In brief, DJ-1 (400 μM) was incubated with 180 mM H_2_O_2_ at 37 °C in 20 mM phosphate buffer (pH 7.4) and 100 mM NaCl for 30 min for generation of complete oxidation species (Cys106-SO_3_^−^). Unreacted H_2_O_2_ was removed by multiple washes with 20 mM phosphate buffer (pH 7.4) and 100 mM NaCl in 10 kDa Amicon Ultra. For partial oxidation, DJ-1 (400 μM) was incubated with 2 molar excess of H_2_O_2_ at 4 °C in 20 mM phosphate buffer (pH 7.4) and 100 mM NaCl overnight. The unreacted H_2_O_2_ was removed as explained above. The DJ-1 was treated with 5 mM DTT to keep it in the un-oxidized state. H_2_O_2_ treated DJ-1(1 μg) was diluted in 500 μl of mass spectrometry grade water and desalted using C18 tip (Pierce, Thermo Scientific) following the manufacturer’s instructions. Desalted proteins were mixed with CHCA (1:1) MALDI matrix and spotted on a MALDI plate. For MALDI peptide mass fingerprinting (PMF) and MS/MS analysis, samples were processed in Sciex 5800 MALDI TOF/TOF mass spectrometer in positive ion reflector mode with ion acceleration voltage 25 KV for MS acquisition and 1 KV for MS/MS. The normalized collision energy was set to 35% for precursor ion fragmentation. The MS and MS/MS spectrum were analysed in MASCOT (version 2.3).

### In vitro oligomerisation and fibrillation of α-synuclein

The low molecular weight (LMW) species of α-synuclein was prepared based on established literature with slight modification^52^. The LMW species was used as starting material for oligomerisation and fibrillation. In brief, α-synuclein lyophilised solution was reconstituted in 20 mM Tris HCl pH 7.4 and was treated with 2 M NaOH for 10 min at 4 °C to dissolve any pre-aggregated proteins and then pH was readjusted to 7.4 by dropwise addition of 6 M HCl. The protein solution was passed through 100 kDa Amicon Ultra 15 to remove aggregated proteins. The protein concentration was determined by a spectroscopic method using molar extinction coefficient 5960 M^−1^cm^−1^ at 280 nm (Perkin-Elmer). α-Synuclein (550 μM) in Tris HCl pH 7.4 was incubated at 37 °C without stirring to get oligomeric species. α-Synuclein (300 μM) in Tris HCl pH 7.4 containing 0.1% sodium azide was incubated at 37 °C with stirring at 300 rpm for 3 days to get mature fibrils. The solution was centrifuged at high speed to precipitate the protein fibrils and the fibrils were washed multiple times to remove oligomeric species.

### Thioflavin T assay

The different samples from primary nucleation as well as seeded aggregation (20 μM) were aliquoted at different time points and incubated with 20 μM of ThT for 5 min as described earlier^41^. Three independent measurements were performed for each sample. ThT fluorescence was recorded using Hitachi F-7000, fluorescence spectrophotometer with excitation at 442 nm and emission was recorded at 482 nm^41,53^.

### Atomic force microscopy

For determination of the size and morphology of protein aggregates samples were aliquoted at different time points placed on freshly cleaved mica instantly and then air-dried. The samples were washed with distilled water to get rid of salts. Samples were imaged in AC mode by JPK Nano Wizard III atomic force microscope (JPK instrument, Berlin, Germany). The drive frequency of silicon cantilever was between 300–320 kHz and the scan rate was between 0.8–1 Hz with a spring constant of 13–77 Nm^−1^. The size of different species were measured from the topographic AFM images with JPK software^41,54^. Quantitative Imaging AFM (QI-AFM) and Force spectroscopy were used for elastic property measurement.The force volume stiffness studies were performed using a JPK Nano wizard III microscope (JPK Instruments AG, Berlin, Germany). Analyses were performed using NANOSENSOR qp-BioAC-50 cantilevers (Switzerland), choosing the ones with a nominal spring constant of 0.15 to 0.55 Nm^−1^. Before every experiment, we had calibrated the mechanical properties of the tip using the JPK software by thermal noise method. The stiffness and adhesive property of proteins were estimated in QI imaging mode where AFM tip was placed in fast oscillation over the sample and the deformation of the cantilever was recorded to reconstruct an image formed by a large number of force-distance (FD) curves. Typical images contain up to 256 × 256 pixels. The length of the curves was between 190–300 nm and the imaging speed ranged from 0.8 to 1.5 lines per second. The maximum applied force was between 2 and 5 nN. Depending on the size and on the resolution of the image, the stiffness of each molecule was measured on a minimum of 15–28 points. Data were processed in a semi-automated way with the JPK data-processing software assuming that the cantilever behaved accordingly to the Hooke’s law. Young’s modulus was calculated assuming the conical tip shape of the cantilever. The shape of each indentation curve was then used to calculate the mechanical properties of the sample such as stiffness, adhesion, and force of dissipation^55–57^.

The imaging of surface property of SH-SY5Y cells were carried out by JPK Nano Wizard III atomic force microscope (JPK instrument, Berlin, Germany), which was equipped with AFM scanner and Zeiss optical microscope. The AFM samples were prepared by fixing the cells with 4% PFA for 20 min. The fixed cells were washed thrice with water. The fixed SH-SY5Y cell images were measured with gold-coated Hydra cantilever in contact mode (APPNANO, USA) with the resonance frequency of 17 kHz ± 4 kHz, force constant of 0.1 Nm^−1^, cantilever length of 200 μm,cantilever width of 40 μm, cantilever thickness of 0.6 μm, tip radius of <10 nm, and tip height of 4–6 μm. All images were taken at a resolution of 256 × 256 or 512 × 512 pixels with a scan speed of 0.8–1 line/s. Image processing and data analysis were performed using JPK software^41,58^.

### Dynamic light scattering

DLS measurements of DJ-1 (1 mg/ml) and its different oxidized state were performed directly on a Zetasizer Nano ZS (Malvern Instruments) which probed scattered light at 173° and used a 630 nm light source. The instrument was equipped with a Peltier temperature controller which was set at 22 °C. Disposable micro-cuvettes were used for size measurements. Every sample was measured ten times^36,41^.

### Circular dichroism spectroscopy

Different samples were diluted with 50 mM phosphate buffer, pH 7.2 to reach a final concentration of 10 µM. Circular dichroism spectra were recorded from 190 to 260 nm wavelength range using JASCO J815 CD spectrophotometer. The spectra were analyzed by DICHROWEB server for secondary structure content.

### ANS binding assay

ANS (8 anilino-1-naphthalene sulfonic acid) (20 μM) was mixed with 10 μM of differentially oxidized DJ-1 in 50 mM Tris-HCl, 150 mM NaCl, pH 7.4 at room temperature. The ANS fluorescence was monitored at different time intervals using Hitachi F-7000, fluorescence spectrophotometer. The excitation wavelength was 372 nm and the emission wavelength scan was carried out between 400 to 600 nm^59^.

### Seeded aggregation

LMW α-synuclein (500 µM) was placed in an incubator at 37 °C with 300 rpm rotation for 72 h. The concentration of LMW α-synuclein in supernatant was estimated after 72 h of aggregation. Subtracting amount of supernatant α-synuclein with initial concentration gives the exact amount of LMW converted to fibrils. Two hundred micromolar of fibrils were sonicated for 30 min in bath sonicator at 50 amplitude. The quality of fibrils and the seed formed were accessed using AFM.

### Binding assay by microscale thermophoresis

Binding assays were carried out on wild type protein using MST performed on a Monolith NT instrument (NanoTemper Technologies). A range of concentrations (0 μM to from 12 μM) of DJ1 variants (DJ-1_Pox_, DJ-1_Cox_, and DJ-1_Uox_) were incubated with red fluorescent dye NT-647 (N-hydroxy succinimide) labelled 500 nM of α-synuclein fibers in 1X PBS supplemented with 1% of Tween 20 for 5 min prior to measurements. The samples were loaded into NanoTemper Technologies glass capillaries and MST measurements were carried out using 80% LED power and high MST power. The dissociation constants (K_d_) were determined using the mass action equation via the NanoTemper Technologies software from duplicate reads of duplicate experiments and reported as ±SD^60^.

### Fiber disintegration assay

The α-synuclein fibrils were prepared as mentioned earlier. Fluorescein isothiocyanate isomer I (Sigma) were used to label α-synuclein fibrils as per manufacturer’s protocol. Briefly, α-synuclein fibrils approx. (50 μM) were diluted in 0.1 M sodium carbonate buffer, pH 9 and treated with 30 molar excess of FITC for 2 h at room temperature. The unreacted FITC were cleared using 3 kDa, 0.5 ml Amicon Ultra and the labelled fibers were incubated with different forms of DJ-1 for 120 h. After incubation, the samples were centrifuged at 16,000 rpm for 45 mins and the resulted pellet was washed twice. The washed pellet was dissolved in 8 M guanidium hydrochloride and the FITC fluorescence was recorded using Spectramax M5, fluorescence spectrophotometer with excitation at 490 nm and emission at 518 nm.

### Annexin V-FITC/ PI assay

SH-SY5Y cells were grown in DMEM Glutamax containing 10% FBS (GIBCO, Thermo Fisher Scientific USA), antibiotics (Penicillin and streptomycin 1%) in a humidified 5% CO_2_ atmosphere at 37 °C and then cells were differentiated with 10 μM of retinoic acid for 3 days^61^. The samples were processed as described earlier^41^. Briefly, the differentiated SH-SY5Y cells were harvested after 48 h of treatment with different preparations of α-synuclein (10 µM) species along with remodeled fibrils samples using 1X PBS with 5 mM EDTA. The cells were pelleted by centrifugation (1200 rpm, 5 minutes) and suspended in binding buffer 0.01 M HEPES (pH 7.4), 0.14 M NaCl, and 2.5 mM CaCl_2_. The cell death assay was performed using the Annexin V-FITC apoptosis detection kit (Sigma, USA). Different samples (1 × 10^6^ cells/ml) were treated with Annexin V-FITC (5 µl) and PI (10 µl) and cells were incubated at room temperature for exactly 15 min in dark. Labelled cells were then sorted by BD FACS verse Flow Cytometer (BD Biosciences) with a minimum of 10,000 events recorded per sample. The data was analyzed in BD Cell Quest Pro software. **P, ***P, and ****P < 0.1, comparisons between groups were analyzed by using one-way ANOVA with Bonferroni’s post-hoc test. Experiments were repeated three times.

### Confocal microscopy

SH-SY5Y cells were labeled with phalloidin conjugated with TRITC for 30 min and washed with 1X PBS thrice. The nucleus of the cells were labeled with DAPI (4’, 6-Diamidino-2-phenylindole, dihydrochloride, and 2 mg/ml). The cells were mounted with prolong gold and dried overnight. All the fluorescence images were taken as Z-stacks on a confocal microscope (Leica SP5, Germany). Control and experimental samples were imaged with the same laser setting and Z-stack thickness. The data were processed using LAS AF Lite software.

### FACS based NO detection

The cellular nitric oxide level was measured by DAF FM DA dye as described earlier^41^. Briefly, different samples were collected in 1X PBS with 5 mM EDTA. The cells were centrifuged at 1200 rcf and resuspended with 10 μM DAF-FM diacetate for 30 min at room temperature in dark. The residual dye was washed with PBS and analyzed for DAF FM-associated fluorescence in a FACS verse flow cytometer in the green channel. The percent of cells showing DAF FM fluorescence were analysed using FlowJo (Tree Star Inc, Ashland, OR, USA) software.

### Internalisation of FITC labelled α-synuclein

The α-synuclein fibrils were prepared as mentioned earlier. Fluorescein isothiocyanate isomer I (Sigma) was used to label α-synuclein fibrils as per manufacturer’s protocol. Briefly, α-synuclein fibrils approx. (50 μM) was diluted in 0.1 M sodium carbonate buffer, pH 9 and treated with 30 molar excess of FITC for 2 h at room temperature. The unreacted FITC was cleared using 3 kDa, 0.5 ml Amicon Ultra and the labelled fibers were used for further experiments. The DJ-1_Pox_ treated α-synuclein after 120 h and labelled α-synuclein alone were exposed to SH-SY5Y cells. The confocal imaging was performed for both samples with the excitation of FITC dye at 488 nm.

### Statistics and reproducibility

Data were expressed as mean ± SD from three to four different experiments and were analyzed by two-tailed unpaired Student’s *t*-test, one-way and two-way ANOVA. Statistical significance was assessed at *p < 0.05, **p < 0.01, ***p < 0.01, and ****p < 0.0001.

### Reporting summary

Further information on research design is available in the Nature Research Reporting Summary linked to this article.

## Supplementary information

Supplementary Information

Description of additional supplementary items

Supplementary Data

Reporting Summary

## Data Availability

AFM images are uploaded in Figshare with following DOIs. Figure 1 10.6084/m9.figshare.9032654.v1, Figure 2 10.6084/m9.figshare.9089348.v1, Figure 3 10.6084/m9.figshare.9089555.v1, Figure 5 10.6084/m9.figshare.9092933.v1, Figure 7 10.6084/m9.figshare.9094277.v1, Figure 9 10.6084/m9.figshare.9095579.v1, Supplementary Fig. 6 10.6084/m9.figshare.9097364.v1, Supplementary Fig. 7 10.6084/m9.figshare.9097394.v1, Supplementary Fig. 9 10.6084/m9.figshare.9097766.v1, Supplemenatary Data 10.6084/m9.figshare.9900431, Source data ara available as Supplementary Data. All other data that support the findings of this study are available from the corresponding author on reasonable request.
