# Supplementary material for: Partially oxidized DJ-1 inhibits α-synuclein nucleation and remodels mature α-synuclein fibrils in vitro
Source: Commun Biol. 2019 Oct 30;2:395. doi: 10.1038/s42003-019-0644-7 (PMC6821844; doi:10.1038/s42003-019-0644-7)
Supplement: Supplementary file 1 — Supplementary Information [file 42003_2019_644_MOESM1_ESM.pdf]

Supplementary Information

Supplementary Methods

Expression and purification of untagged DJ-1

Recombinant pET3a-DJ-1 (Addgene) construct containing full length human DJ-1 gene was transformed into BL21(DE3) *E. coli* cells and *E. coli* cells were grown in LB media at 37 ºC until OD at 600 mm reached to 0.8. The cells were then induced with 0.5 mM of IPTG at 37 ºC for another 4 h. The cells were harvested by centrifuging and cells pellet was resuspended in 20 mM. The resuspended cells were homogenized and the lysate was centrifuged at 13000 x g for 60 min. The clear supernatant was treated with 60 % ammonium sulphate. Ammonium sulphate precipitated proteins were removed by centrifugation at 10000 x g for 30 min and the clear supernatant was loaded on to HiTrap Phenyl FF (GE Healthcare) column. The column was washed with 25 mM phosphate buffer of pH 7.0 containing 60 % ammonium sulphate. DJ-1 protein was eluted with 25 mM phosphate buffer of pH 7.0 and finally, the protein was further purified in a HiPrep Sephacryl S-200 HR (GE Healthcare column). The purity of the protein was checked in SDS-PAGE and the intact mass of DJ-1 was confirmed by MALDI-TOF mass spectrometry.

Degylcase activity of untagged DJ-1 and 6xHis-DJ-1.

Reduced glutathione was incubated with methylglyoxal (1: 0.9 molar ratio) at 25 ºC to form glycated conjugate of methylglyoxal and it is the substrate of DJ-1. The concentration of MGO adduct of GSH was estimated using the molar extinction coefficient at 280 nm = 922 M^{-1} cm^{-1}. The deglycase activity was estimated by monitoring the loss of absorbance at 288 nm with time. Varying substrate concentrations (2 mM to 12 mM) were incubated with DJ-1 (without tag and

6xHis tag) in a final concentration of 20 µM in a 96 well plate and enzymatic activity was monitored. Michaelis-Menten plot and Lineweaver-Burk plot of respective 6xHis tag-DJ-1 and DJ-1 (without tag) were estimated by using GraphPad Prism software. The K_m, k_{cat} and k_{cat}/K_m were calculated for both 6xHis-tagged DJ-1 and DJ-1 (without tag).

Supplementary Figures

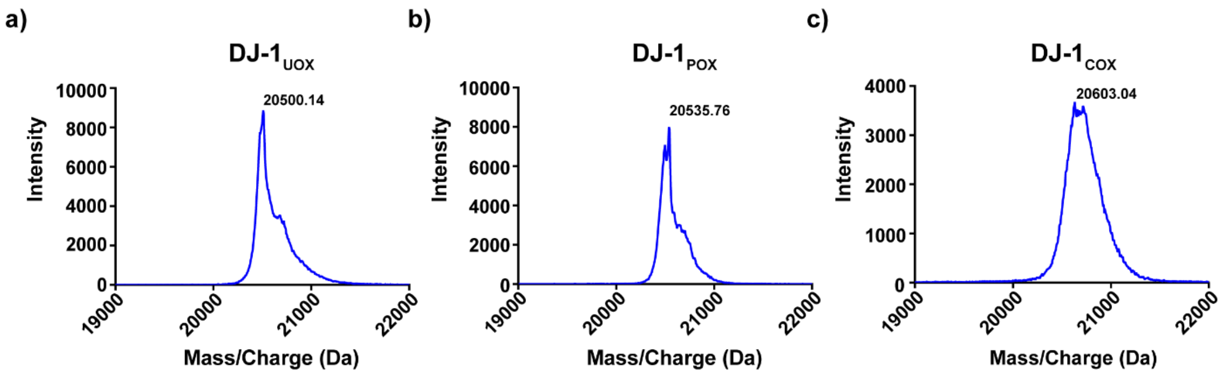

Supplementary Figure 1. MALDI-TOF of different forms of DJ-1. (a) DJ-1_{UOX} (b) DJ-1_{POX} (c) DJ-1_{COX}

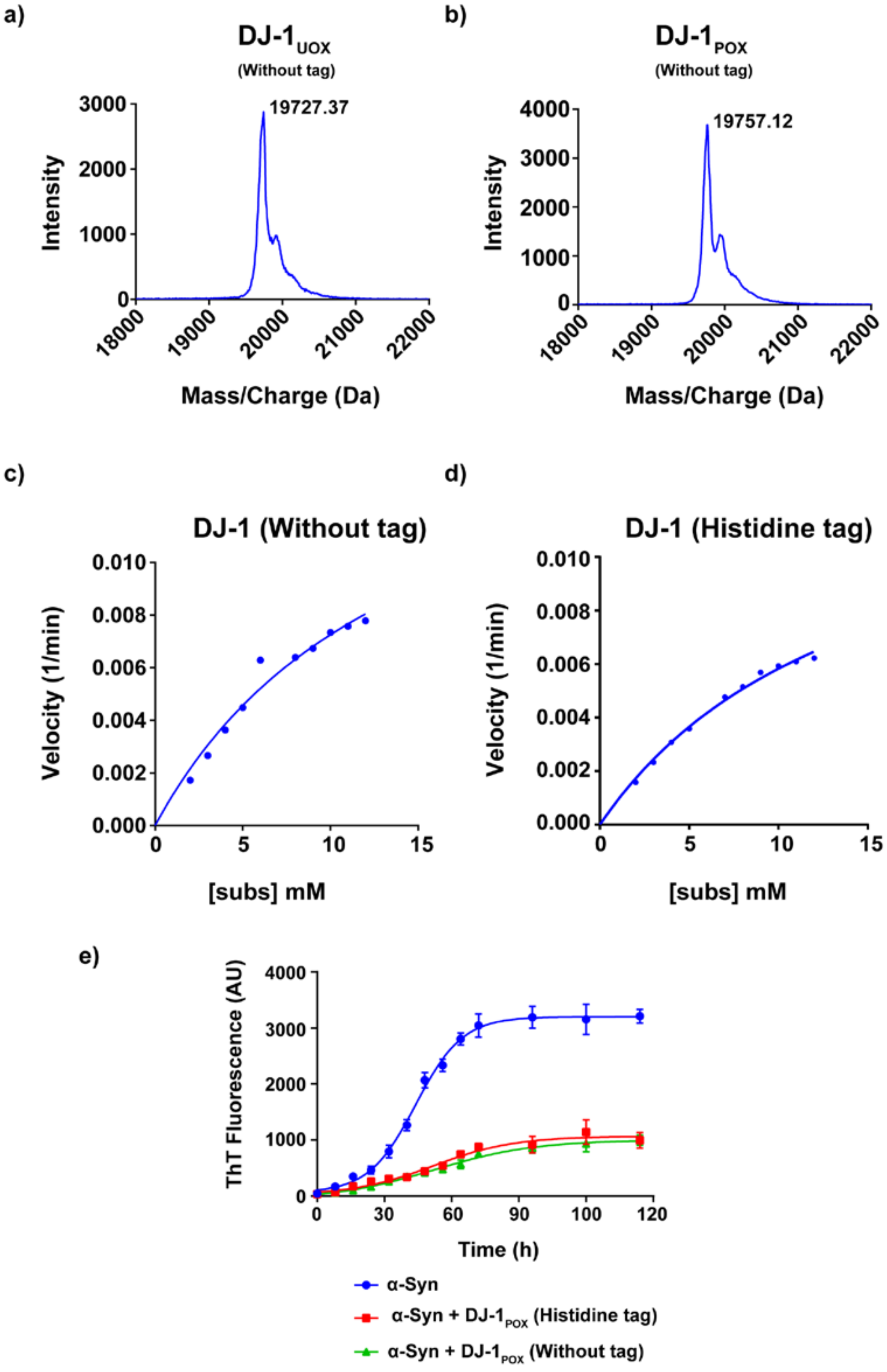

Supplementary Figure 2. MALDI-TOF of un-oxidized and oxidized forms of untagged DJ-1, deglycase activity and aggregation assay. *(a) untagged DJ-1$_{Uox}$, (b) untagged DJ-1$_{Pox}$, (c and d) deglycase activity (e) aggregation kinetics of α-synuclein with untagged DJ-1$_{Uox}$ and taged DJ-1$_{Pox}$.*

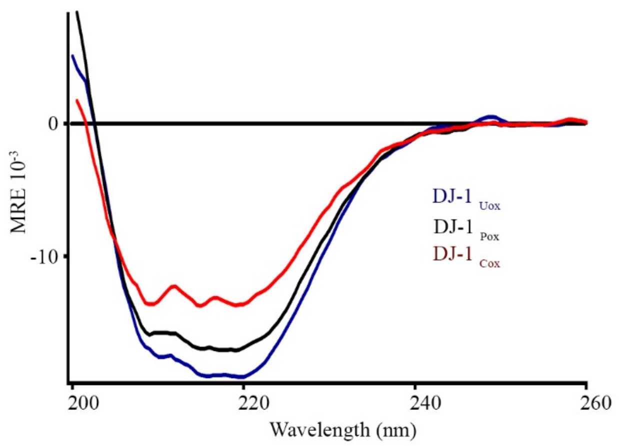

Supplementary Figure 3. CD Spectroscopy. *The spectra of different αsynuclein variants were recorded from 190 nm to 260 nm using JASCO 815 CD spectrometer.*

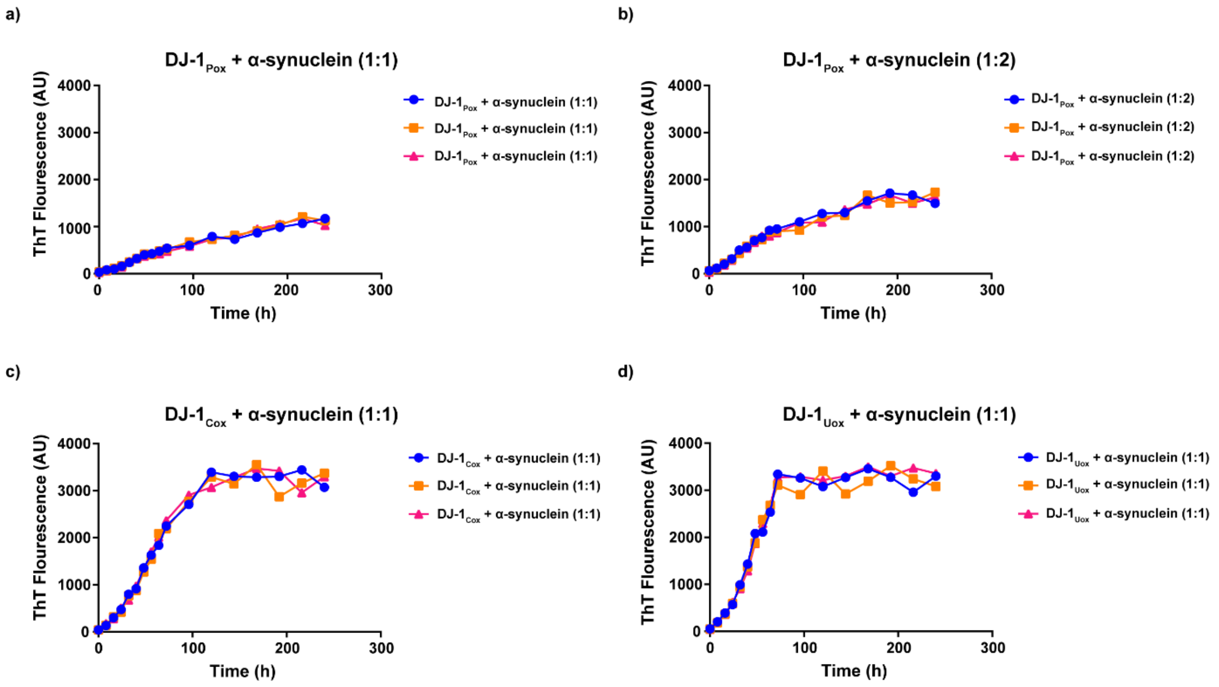

Supplementary Figure 4. Reproducibility experiment **DJ-1$_{Pox}$** ***inhibits nucleation of α-synuclein.*** *(a) Time-dependent visualization of α-synuclein aggregation inhibition in the presence of* DJ-1$_{Pox}$, DJ-1$_{Uox,}$ and DJ-1$_{Cox.}$

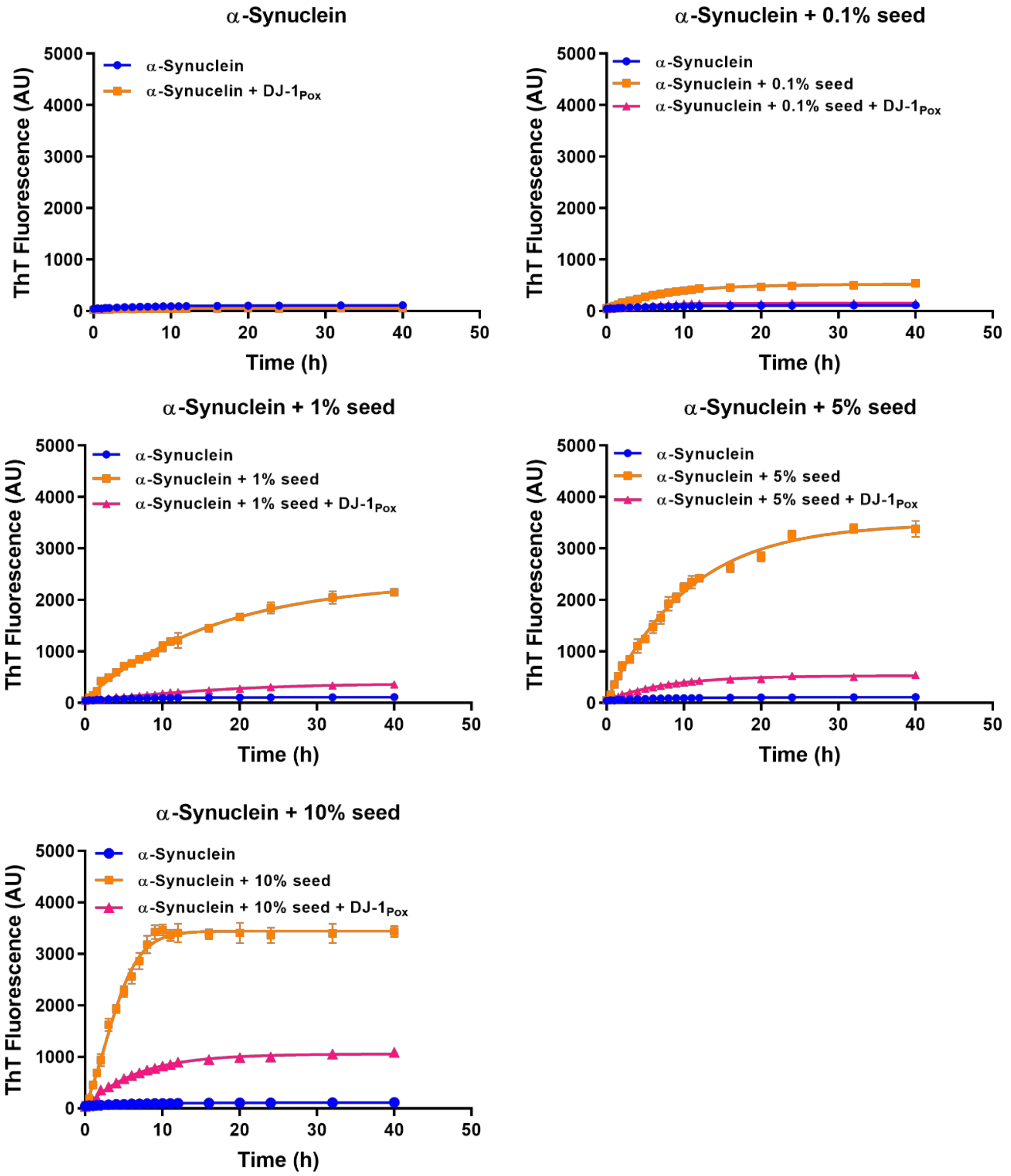

***Supplementary Figure 5. Seeded aggregation of α-synuclein in the presence of* **$DJ\text{-}1_{Pox}$**.** *The increase of relative aggregate mass upon addition of free monomers (50 µM) of α-synuclein to existing seeds (0.1 %, 1 %, 5 % and 10 %) was measured over time by Thioflavin T fluorescence assay.*

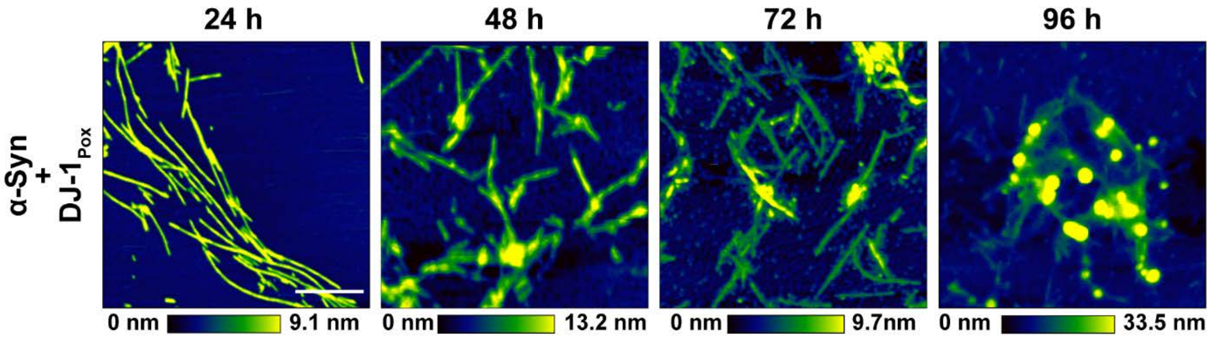

***Supplementary Figure 6. Remodeling of mature α-synuclein fibrils in the presence of* DJ-1$_{Pox}$.** *AFM images showing α-synuclein mature fibrils with DJ-1$_{Pox}$ at different time points. (Scale bar represents 500 nm)*

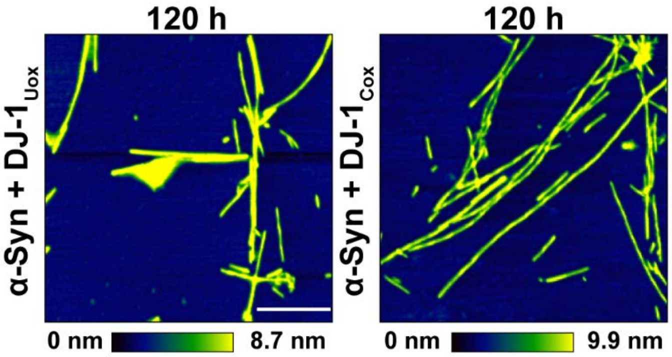

***Supplementary Figure 7. Mature α-synuclein fibrils in the presence of* DJ-1$_{\mathbf{Uox\ and}}$ DJ-1$_{\mathbf{Cox}}$.**
AFM images showing (a) α-synuclein mature fibrils with DJ-1$_{\mathrm{Uox}}$ *(b) α-synuclein mature fibrils with* DJ-1$_{\mathrm{Cox}}$. *(Scale bar represents 500 nm)*

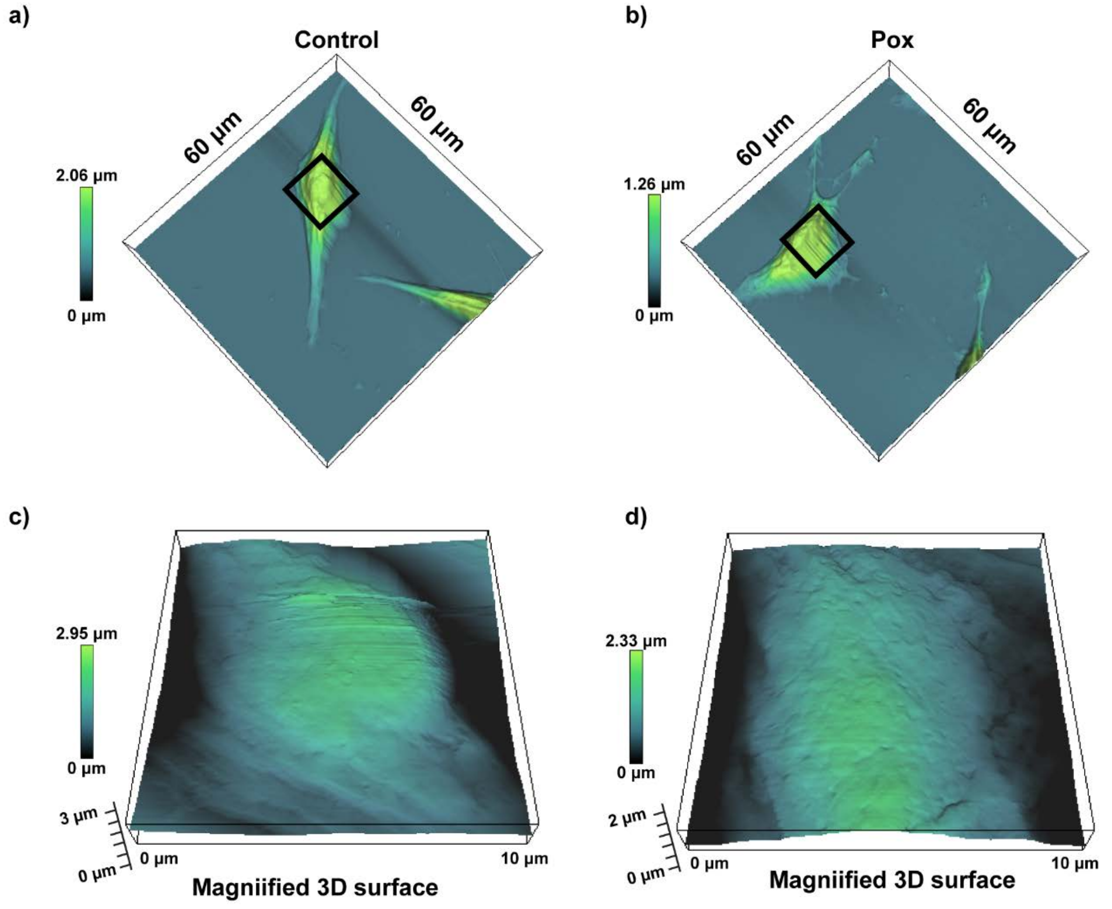

Supplementary Figure 8. Membrane architecture in the presence of BSA and DJ-1$_{Pox}$. (*a) and (b) represents 3D atomic force microscopic images (60µm x 60µm) of SH-SY5Y cells. Figure (c) and (d) represent a magnified 3D surface (10µm x 10µm). (a, c) BSA (b, d) DJ-1$_{Pox}$.*

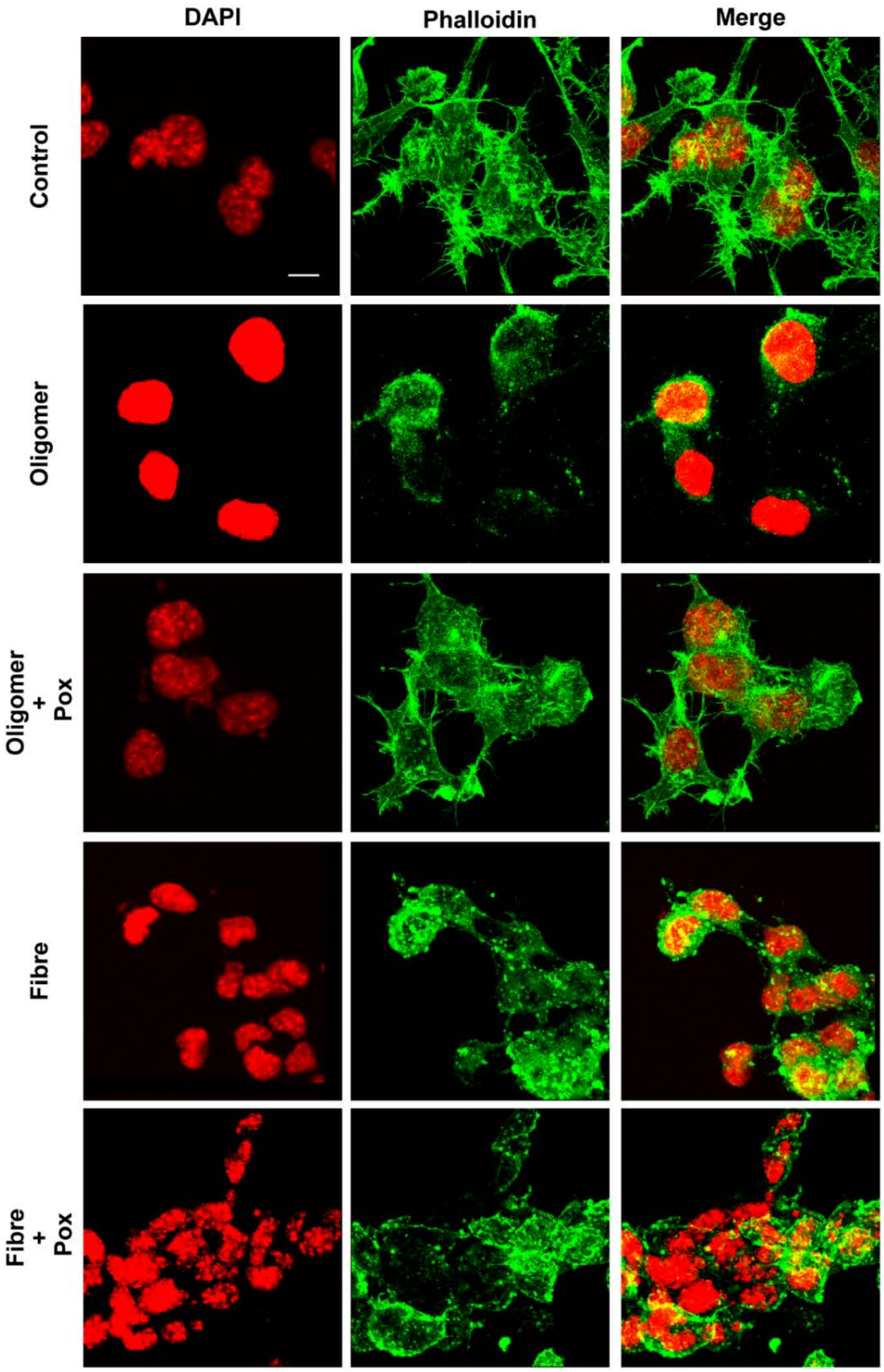

Supplementary Figure 9. Distribution of actin in response to treatment of α- synuclein and DJ1$_{Pox}$. *Actin was visualized by FITC-labeled phalloidin (green) and nucleus stained with DAPI (Red). Distinct actin spikes were seen in control and oligomer with DJ1$_{Pox}$ treated cells. The retraction of actin to the perinuclear region is seen in cells treated with oligomer, fibrils and fibrils with DJ1$_{Pox}$ treated cells. (Scale bar represents 10 nm)*

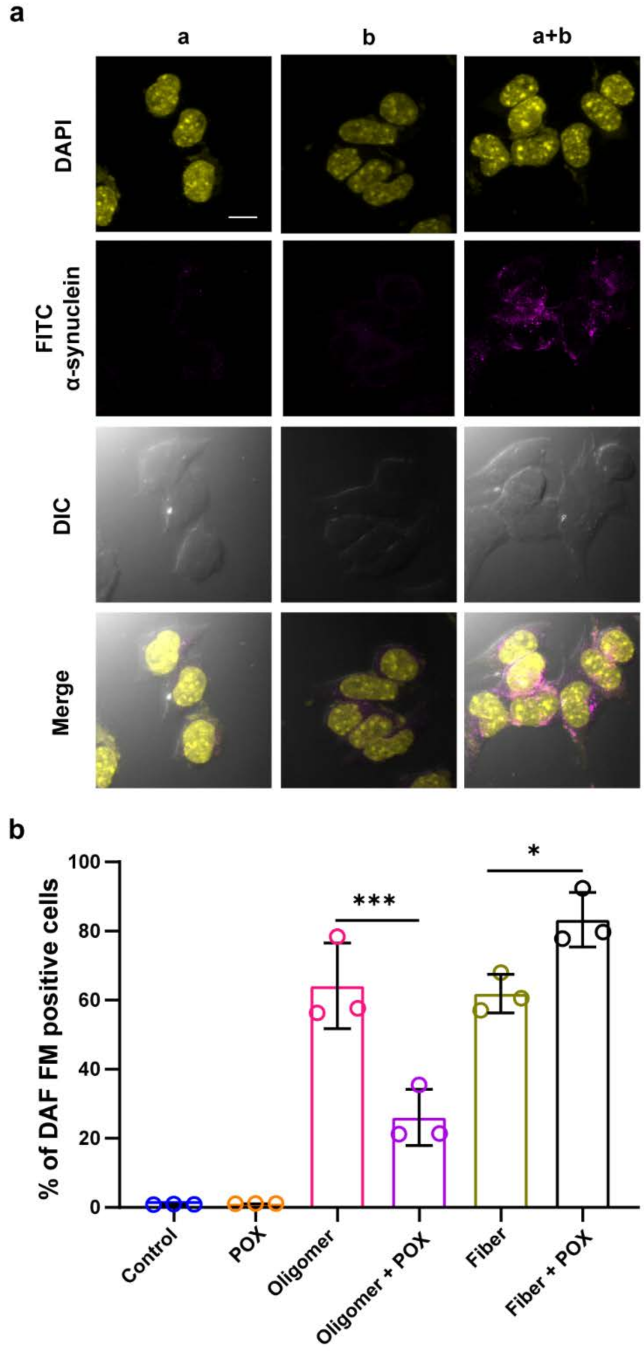

Supplementary Figure 10. (a) Internalisation of DJ-1$_{Pox}$ remodeled α-synuclein fibrils (b) Nitric oxide release assay. *DAF-FM diacetate, a cell-permeable indicator, was used to monitor nitric oxide release in monomer and α- synuclein oligomeric variants treated SH-SY5Y cells and in control cells. Values are mean ± s.e.m., n ≥ 3 (Scale bar represents 10 nm)*

Supplementary Table 1. MS analysis of trypsin peptide fragment of oxidized DJ-1

**Peptide sequence**	**Peptide sequence**	**Mass**		
		**DJ-1$_{Uox}$**	**DJ-1$_{Pox}$**	**DJ-1$_{Cox}$**
**13-27**	**GAEEMETVIPVDVMR**	**1691.8, 1O added**	**1691.8, 1O added**	**1707.8, 2O added**
**33-48**	VTVAGLAGKDPVQCSR	Not detected	1600.8	1600.8
**64-89**	EGPYDVVVLPGGNLGAQNL SESAAVK	2584.3	2584.3	2584.3
**90-98**	EILKEQENR	1158.6	1158.6	1158.6
**100–122**	**GLIAAICAGPTALLAHEIGF GSK**	**2210.2**	**2242.2, 2O added**	**2258.2, 3O added**
**123-130**	VTTHPLAK	866.5	866.5	866.5
**133-145**	**MMNGGHYTYSENR**	**1576.6, 1O added**	**1576.6, 1O added**	**1592.6, 2O added**
**149-156**	DGLILTSR	874.5	874.5	874.5
**157-175**	GPGTSFEFALAIVEALNGK	1922.0	1922.0	1922.0

**Peptide sequence**	**Peptide sequence**	**Mass**		
		**DJ-1$_{Uox}$**	**DJ-1$_{Pox}$**	**DJ-1$_{Cox}$**
**13-27**	**GAEEMETVIPVDVMR**	**1691.8, 1O added**	**1691.8, 1O added**	**1707.8, 2O added**
**33-48**	VTVAGLAGKDPVQCSR	Not detected	1600.8	1600.8
**64-89**	EGPYDVVVLPGGNLGAQNL SESAAVK	2584.3	2584.3	2584.3
**90-98**	EILKEQENR	1158.6	1158.6	1158.6
**100–122**	**GLIAAICAGPTALLAHEIGF GSK**	**2210.2**	**2242.2, 2O added**	**2258.2, 3O added**
**123-130**	VTTHPLAK	866.5	866.5	866.5
**133-145**	**MMNGGHYTYSENR**	**1576.6, 1O added**	**1576.6, 1O added**	**1592.6, 2O added**
**149-156**	DGLILTSR	874.5	874.5	874.5
**157-175**	GPGTSFEFALAIVEALNGK	1922.0	1922.0	1922.0

Supplementary Table 2. K_m and k_{cat} of His-tag and without tagged DJ-1

DJ-1 without tag)

K_m (M)	k_{cat} (s^{-1})	k_{cat}/K_m ($M^{-1}s^{-1}$)
**0.014**	**0.01**	**1.02**

DJ-1 (histidine tag)

K_m (M)	k_{cat} (s^{-1})	k_{cat}/K_m ($M^{-1}s^{-1}$)
**0.014**	**0.01**	**0.082**

Supplementary Table 3. The lag time of aggregation (t_{lag}) and apparent rate constants (K_{app}) for a different group of α-synuclein with DJ-1 in Thioflavin T kinetics.

**Mutants**	**α-Syn**	**$DJ\text{-}1_{Pox}$ + α-Syn (1:1)**	**$DJ\text{-}1_{Pox}$ + α-Syn (1:2)**	**$DJ\text{-}1_{Cox}$ + α-Syn (1:1)**	**$DJ\text{-}1_{Uox}$ + α-Syn (1:1)**
**t_{lag} (h)**	27.63±0.85	35.13±1.60	25.84±1.91	31.46±0.60	27.21±0.77
**K_{app} (h^{-1})**	0.055±0.006	0.016±0.0003	0.023±0.001	0.034±0.0015	0.051±0.0012
