# Supplementary material for: Partially oxidized DJ-1 inhibits α-synuclein nucleation and remodels mature α-synuclein fibrils in vitro
Source: Commun Biol. 2019 Oct 30;2:395. doi: 10.1038/s42003-019-0644-7 (PMC6821844; doi:10.1038/s42003-019-0644-7)
Supplement: Supplementary file 2 — Description of additional supplementary items [file 42003_2019_644_MOESM2_ESM.docx]

Description of additional supplementary items

Supplementary Data : Raw data-points underlying the graphs
