# Supplementary material for: Partially oxidized DJ-1 inhibits α-synuclein nucleation and remodels mature α-synuclein fibrils in vitro
Source: Commun Biol. 2019 Oct 30;2:395. doi: 10.1038/s42003-019-0644-7 (PMC6821844; doi:10.1038/s42003-019-0644-7)
Supplement: Supplementary file 4 — Reporting Summary [file 42003_2019_644_MOESM4_ESM.pdf]

nature research

Corresponding author(s): Dr. Tushar Kanti Maiti

Reporting Summary

Nature Research wishes to improve the reproducibility of the work that we publish. This form provides structure for consistency and transparency in reporting. For further information on Nature Research policies, see Authors & Referees and the Editorial Policy Checklist.

Statistical parameters

When statistical analyses are reported, confirm that the following items are present in the relevant location (e.g. figure legend, table legend, main text, or Methods section).

n/a	Confirmed	
☐	☒	The exact sample size (n) for each experimental group/condition, given as a discrete number and unit of measurement
☐	☒	An indication of whether measurements were taken from distinct samples or whether the same sample was measured repeatedly
☐	☒	The statistical test(s) used AND whether they are one- or two-sided<br>*Only common tests should be described solely by name; describe more complex techniques in the Methods section.*
☒	☐	A description of all covariates tested
☒	☐	A description of any assumptions or corrections, such as tests of normality and adjustment for multiple comparisons
☐	☒	A full description of the statistics including central tendency (e.g. means) or other basic estimates (e.g. regression coefficient) AND variation (e.g. standard deviation) or associated estimates of uncertainty (e.g. confidence intervals)
☐	☒	For null hypothesis testing, the test statistic (e.g. F, t, r) with confidence intervals, effect sizes, degrees of freedom and P value noted<br>*Give P values as exact values whenever suitable.*
☒	☐	For Bayesian analysis, information on the choice of priors and Markov chain Monte Carlo settings
☒	☐	For hierarchical and complex designs, identification of the appropriate level for tests and full reporting of outcomes
☒	☐	Estimates of effect sizes (e.g. Cohen's d, Pearson's r), indicating how they were calculated
☐	☒	Clearly defined error bars<br>*State explicitly what error bars represent (e.g. SD, SE, CI)*

Our web collection on statistics for biologists may be useful.

Software and code

Policy information about availability of computer code

Data collection	n/a
Data analysis	Graph Pad Prism 7<br>JPK SPM Data processing Software<br>Leica Application Suite Advanced Fluorescence (LAS AF) Software<br>FlowJo (Tree Star Inc, Ashland, OR, USA) software<br>BD Cell Quest Pro software

For manuscripts utilizing custom algorithms or software that are central to the research but not yet described in published literature, software must be made available to editors/reviewers upon request. We strongly encourage code deposition in a community repository (e.g. GitHub). See the Nature Research guidelines for submitting code & software for further information.

Data

Policy information about availability of data

All manuscripts must include a data availability statement. This statement should provide the following information, where applicable:

- Accession codes, unique identifiers, or web links for publicly available datasets
- A list of figures that have associated raw data
- A description of any restrictions on data availability

The data that support the findings of this study are available on request. AFM images are uploaded in Figshare with following DOI.
Figure 1 https://doi.org/10.6084/m9.figshare.9032654.v1
Figure 2 https://doi.org/10.6084/m9.figshare.9089348.v1
Figure 3 https://doi.org/10.6084/m9.figshare.9089555.v1
Figure 5 https://doi.org/10.6084/m9.figshare.9092933.v1
Figure 7 https://doi.org/10.6084/m9.figshare.9094277.v1
Figure 9 https://doi.org/10.6084/m9.figshare.9095579.v1
Supplementary Figure 6 https://doi.org/10.6084/m9.figshare.9097364.v1
Supplementary Figure 7 https://doi.org/10.6084/m9.figshare.9097394.v1
Supplementary Figure 9 https://doi.org/10.6084/m9.figshare.9097766.v1

Field-specific reporting

Please select the best fit for your research. If you are not sure, read the appropriate sections before making your selection.

☒ Life sciences ☐ Behavioural & social sciences ☐ Ecological, evolutionary & environmental sciences

For a reference copy of the document with all sections, see nature.com/authors/policies/ReportingSummary-flat.pdf

Life sciences study design

All studies must disclose on these points even when the disclosure is negative.

Sample size	No sample-size calculations were performed. Sample size was determined to be adequate based on the magnitude and consistency of measurable differences between groups.
Data exclusions	On principle, data were only excluded for failed experiments for example cell contamination.
Replication	Replicate experiments were statistical significant.
Randomization	Elasticity measurement were performed randomly to experimental and control groups.
Blinding	Investigators were not blinded in any experiment.

Reporting for specific materials, systems and methods

Materials & experimental systems

n/a	Involved in the study	
☒	☐	Unique biological materials
☒	☐	Antibodies
☐	☒	Eukaryotic cell lines
☒	☐	Palaeontology
☒	☐	Animals and other organisms
☒	☐	Human research participants

Methods

n/a	Involved in the study	
☒	☐	ChIP-seq
☐	☒	Flow cytometry
☒	☐	MRI-based neuroimaging

Eukaryotic cell lines

Policy information about cell lines

Cell line source(s)	ATCC SH-SY5Y cells.
Authentication	SH-SY5Y cells was a kind gift from Dr. Ranjit Giri, National Brain Research Centre, Manesar, Haryana, India.
Mycoplasma contamination	Cell line is negative for mycoplasma.
Commonly misidentified lines (See ICLAC register)	No cell lines used are listed in the database of commonly misidentified cell lines.

Flow Cytometry

Plots

Confirm that:

- [x] The axis labels state the marker and fluorochrome used (e.g. CD4-FITC).
- [x] The axis scales are clearly visible. Include numbers along axes only for bottom left plot of group (a 'group' is an analysis of identical markers).
- [x] All plots are contour plots with outliers or pseudocolor plots.
- [x] A numerical value for number of cells or percentage (with statistics) is provided.

Methodology

Sample preparation	Sample preparation listed in Methods
Instrument	FACS Verse
Software	BD Cell Quest Pro software
Cell population abundance	10,000-30,000 cells
Gating strategy	Cells were gated on the basis of size and granularity. Percent positive cells for Annexin and PI as well as mean fluorescence intensity for DAF fluorescence was calculated as required.

- [x] Tick this box to confirm that a figure exemplifying the gating strategy is provided in the Supplementary Information.
